# Cytotoxic Effects of ZnO and Ag Nanoparticles Synthesized in Microalgae Extracts on PC12 Cells

**DOI:** 10.3390/md22120549

**Published:** 2024-12-04

**Authors:** Giacomo Fais, Agnieszka Sidorowicz, Giovanni Perra, Debora Dessì, Francesco Loy, Nicola Lai, Paolo Follesa, Roberto Orrù, Giacomo Cao, Alessandro Concas

**Affiliations:** 1 Interdepartmental Centre of Environmental Science and Engineering (CINSA), University of Cagliari, Via San Giorgio 12, 09124 Cagliari, Italy; giacomo.fais@unica.it (G.F.); sid.agnieszka@gmail.com (A.S.); giovanni.perra@unica.it (G.P.); nicola.lai@unica.it (N.L.); giacomo.cao@unica.it (G.C.); 2 Department of Mechanical, Chemical and Materials Engineering, University of Cagliari, Via Marengo 2, 09123 Cagliari, Italy; 3 Department of Life and Environmental Sciences, University of Cagliari, Cittadella Universitaria di Monserrato, Monserrato, 09042 Cagliari, Italy; deboradessi95@gmail.com (D.D.); paolo.follesa@unica.it (P.F.); 4 Department of Biomedical Sciences, University of Cagliari, Cittadella Universitaria di Monserrato, Monserrato, 09042 Cagliari, Italy; floy@unica.it; 5 Center for Advanced Studies, Research and Development in Sardinia (CRS4), Loc. Piscina Manna, Building 1, 09050 Pula, Italy

**Keywords:** green synthesis, *Chlorella vulgaris*, silver nanoparticles (Ag NPs), zinc oxide nanoparticles (ZnO NPs), PC12 cells, microalgae, mitochondria, bioenergetics

## Abstract

The green synthesis of silver (Ag) and zinc oxide (ZnO) nanoparticles (NPs), as well as Ag/Ag_2_O/ZnO nanocomposites (NCs), using polar and apolar extracts of *Chlorella vulgaris*, offers a sustainable method for producing nanomaterials with tunable properties. The impact of the synthesis environment and the nanomaterials’ characteristics on cytotoxicity was evaluated by examining reactive species production and their effects on mitochondrial bioenergetic functions. Cytotoxicity assays on PC12 cells, a cell line originated from a rat pheochromocytoma, an adrenal medulla tumor, demonstrated that Ag/Ag_2_O NPs synthesized with apolar (Ag/Ag_2_O NPs A) and polar (Ag/Ag_2_O NPs P) extracts exhibited significant cytotoxic effects, primarily driven by Ag^+^ ion release and the disruption of mitochondrial function. However, it is more likely the organic content, rather than size, influenced anticancer activity, as commercial Ag NPs, despite smaller crystallite sizes, exhibit less effective activity. ZnO NPs P showed increased reactive oxygen species (ROS) generation, correlated with higher cytotoxicity, while ZnO NPs A produced lower ROS levels, resulting in diminished cytotoxic effects. A comparative analysis revealed significant differences in LD_50_ values and toxicity profiles. Differentiated PC12 cells showed higher resistance to ZnO, while AgNPs and Ag/Ag_2_O-based materials had similar effects on both cell types. This study emphasizes the crucial role of the synthesis environment and bioactive compounds from *C. vulgaris* in modulating nanoparticle surface chemistry, ROS generation, and cytotoxicity. The results provide valuable insights for designing safer and more effective nanomaterials for biomedical applications, especially for targeting tumor-like cells, by exploring the relationships between nanoparticle size, polarity, capping agents, and nanocomposite structures.

## 1. Introduction

The increasing demand for sustainable and effective biomedical applications has led to the emergence of nanotechnology as a promising solution, particularly in cancer therapy, where nanoparticles (NPs) offer enhanced precision and reduced side effects. Innovative applications of metal-based NPs have been developed in various fields, including medicine. The potential of metal-based NPs as drug delivery systems, as well as in diagnostic imaging, tissue engineering, and targeted therapies for cancer treatment, has been demonstrated [1,2,3].

NPs offer notable advantages in cancer therapy by improving the precision of treatments. Due to their small size and surface modifications, NPs can be specifically directed to tumor sites, enhancing the delivery of therapies while reducing side effects in surrounding healthy tissues. They can also be designed to respond to specific conditions within the tumor environment, enabling a more controlled and effective release of treatments. Such flexibility makes NPs a valuable tool in addressing common challenges in cancer care, helping to improve the overall success of therapeutic interventions [4,5].

Among the wide range of available NPs, metal-based nanoparticles have gained particular attention due to their impressive physicochemical properties. These include silver nanoparticles (Ag NPs) and zinc oxide nanoparticles (ZnO NPs). While Ag NPs are primarily recognized for their antimicrobial properties, diverse research has shown their potential in cancer therapy and photocatalysis [6]. Ag NPs can generate reactive oxygen species (ROS), which induce oxidative stress in cancer cells, leading to DNA damage and apoptosis [7,8,9,10]. Their ability to disrupt cellular membranes and interfere with essential biological processes makes them highly effective in targeting cancer cells. Furthermore, Ag NPs have shown promise in enhancing the efficacy of chemotherapeutic agents when used in combination with traditional therapies [4,5]. Similarly, zinc oxide nanoparticles (ZnO NPs) have gained recognition for their dual role in both therapeutic and diagnostic applications (theragnostics). ZnO NPs can generate ROS, leading to oxidative stress and cell death in cancer cells. Their ability to interact with the acidic microenvironment of tumors makes them ideal candidates for photodynamic therapy (PDT), where light activation can further enhance their cytotoxic effects. ZnO NPs also possess photoluminescent properties that make them useful as imaging agents for diagnostic purposes [4].

While metal-based nanoparticles have relevant potential, their traditional chemical synthesis methods pose concerns due to the use of toxic reagents, which pose environmental and health risks [11,12]. To address these concerns, recent advancements have emphasized the development of green synthesis techniques, which use biological agents such as plant extracts and microorganisms to produce nanoparticles in an eco-friendly manner. This approach not only reduces toxicity but also improves the biocompatibility and stability of the nanoparticles, making them more suitable for clinical applications [13,14].

Microalgae, particularly *Chlorella vulgaris*, have emerged as an excellent biological platform for synthesizing metal NPs due to their high content of bioactive metabolites, such as proteins, polysaccharides, and lipids, which play a dual role in the reduction and stabilization of metal ions [6,15,16]. These biological components can effectively convert metal salts into NPs under mild conditions, avoiding the need for toxic chemicals and reducing energy consumption. Moreover, microalgae-based NP synthesis offers several advantages, such as scalability and cost-effectiveness, while also presenting the potential for integrating waste valorization by using microalgae biomass grown in wastewater.

Furthermore, the different types of NPs, ZnO NPs, and Ag NPs synthesized through green methods have demonstrated significant cytotoxic effects on various cancer cell lines. ZnO NPs possess multiple functional properties, including high chemical stability, strong UV absorption, and broad-spectrum antimicrobial activity due to their ability to release Zn^2+^ ions and generate ROS [12]. These properties make ZnO NPs ideal candidates for therapeutic applications, such as cancer therapy, where they can act as radiosensitizers, enhancing the effects of radiotherapy on cancer cells [7,12,17,18]. Similarly, Ag NPs have been extensively studied for their biological, antibacterial, and anticancer properties, largely attributed to their ability to release Ag^+^ ions, which disrupt cellular membranes and induce oxidative stress [10,19,20,21].

The neurotoxic effects of metal nanoparticles (NPs) are of particular interest in studies involving neuroendocrine cell lines like adrenal pheochromocytoma, as neural tissues are especially susceptible to oxidative stress [22]. In the context of these findings, PC12 cells, derived from rat pheochromocytoma, represent a cancerous cell line commonly employed as a model for neuronal differentiation and neurobiological research [23,24,25]. This study specifically selected undifferentiated PC12 cells for their neuroendocrine origin, making them particularly relevant for investigating the neurotoxic effects of metal nanoparticles. The PC12 cell line, derived from rat pheochromocytoma, was selected for this study due to its widespread use in investigating neuronal differentiation and cytotoxicity. As a well-established model for assessing oxidative stress and toxic damage at the neuronal level, PC12 cells are particularly sensitive to oxidative stress, making them ideal for studying the neurotoxic effects of metallic nanoparticles like Ag, ZnO, and their nanocomposites, which can disrupt mitochondrial function and induce cytotoxicity. This cell line effectively demonstrates how nanoparticles synthesized using extracts impact undifferentiated cells, which are highly susceptible to toxic agents. As neural tissues are particularly vulnerable to oxidative stress, the use of undifferentiated PC12 cells facilitates the assessment of how ZnO and Ag NPs interact with neural-like environments in their most sensitive state. This focus on undifferentiated cells is crucial for understanding how early-stage cells, which are less specialized and more susceptible to external stressors, respond to potential toxic agents.

**Figure 1:**
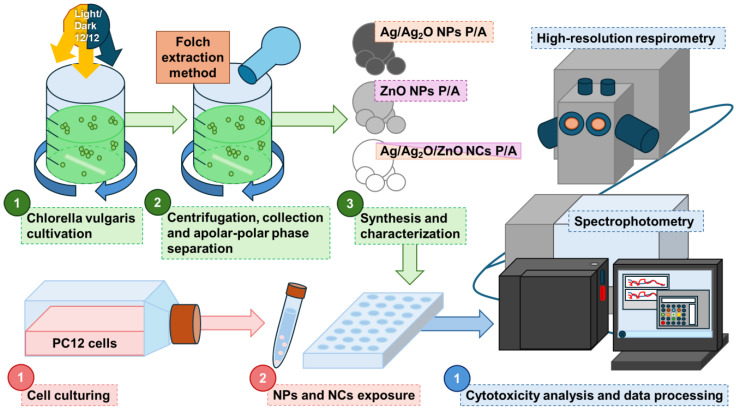
Scheme of the experimental activity involving green synthesis and characterization of metallic NPs using Chlorella vulgaris extracts, as well as tests on PC12 cells using spectrophotometric assays and respirometry analysis.

Several studies have shown that ZnO and Ag NPs effectively induce cytotoxicity in PC12 cells by generating ROS and disrupting mitochondrial function, leading to oxidative stress, reduced ATP production, and apoptosis. Additionally, these nanoparticles impair DNA integrity and affect neuronal differentiation, with their toxicity often depending on size and concentration [22,24,25,26,27,28,29]. Nanomaterials are widely recognized for their potent ability to induce apoptosis in cancer cells. Typically, studies focus on only one type of nanomaterial, often utilizing one specific extract, while here we also focused on nanocomposites. Moreover, to the best of our knowledge, the Folch extraction method has never been employed before for obtaining extracts specifically for nanoparticle synthesis. The existing gap in the research leaves the potential effects of varying phytochemical compositions on nanoparticle properties and performance largely unexamined. This situation underscores the critical importance of investigating how various extracts may influence synthesis and functionality. Moreover, this is the first time that Ag/Ag_2_O NPs, ZnO NPs, and Ag/Ag_2_O/ZnO NC synthesized from *Chlorella vulgaris* extracts have been used for anticancer applications. Building on these properties, our study focuses on the green synthesis of ZnO NPs, Ag NPs, and Zn/Ag hybrid NPs using polar (P) and apolar (A) extracts of the microalgae *C. vulgaris* and investigates their effects on undifferentiated PC12 cells. Harnessing the bioactive compounds in these extracts allows for the development of a sustainable and eco-friendly synthesis method that not only reduces environmental impact, but also enhances the biomedical potential of the nanoparticles. The alternative synthesis method aims to be efficient and scalable, offering various biomedical applications. The cytotoxic effects of green-synthesized NPs on PC12 cells are also investigated, with a focus on assessing their impact on cell viability using the MTT assay. A graphic scheme summarizing the activity carried out in this work is reported in Figure 1.

## 2. Results and Discussion

### 2.1. Phase Composition and Crystallinity of ZnO Nanoparticles Synthesized from Polar and Apolar Extracts

**Figure 2:**
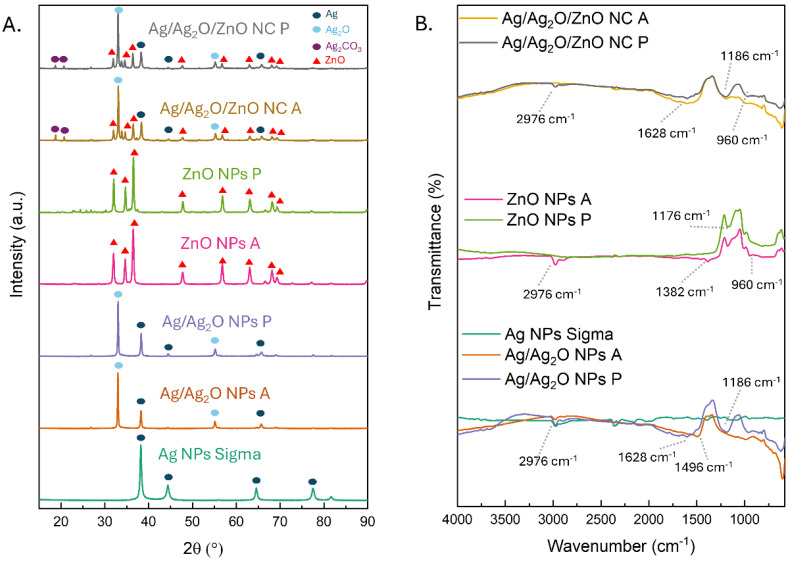
Structural and chemical characterization: (**A**) XRD spectra; (**B**) FTIR analysis.

The phase composition of the obtained materials in comparison with the commercially obtained Ag NPs (Sigma Aldrich SRL, Milano, Italy) was assessed using X-ray diffraction (XRD) analysis (Figure 2A). The XRD pattern of Ag NPs Sigma displays sharp and intense peaks corresponding to the cubic Fm-3m (225) structure of metallic Ag, confirming its high crystallinity and phase purity. The crystallite size was estimated to be around 14.4 nm. The XRD patterns of the NPs synthesized from both polar and apolar extracts exhibited the same diffraction peaks. In the samples, distinct peaks were observed at 2θ values of 38.1°, 44.3°, 64.5°, and 77.4°, corresponding to the (111), (200), (220), and (311) crystallographic planes, respectively (JCPDS Card No. 87-0720). The peaks from the polar and apolar phases also confirm the formation of the cubic Pn-3m (224) Ag_2_O phase at 2θ values of 32.8° and 55.0° attributed to (111) and (220), respectively (JCPDS Card No. 75-1532). The Ag/Ag_2_O NPs P showed a crystallite size of about 24.8 nm. The apolar extract showed the same set of peaks, indicating that the non-polar environment did not significantly alter the crystalline structure of the materials. However, the peak intensities were slightly reduced compared to the polar-extract-derived samples, resulting in an increased crystallite size of approximately 30.7 nm, suggesting a difference in crystallinity or particle size, likely due to the distinct stabilization mechanisms in the two phases [30].

For the ZnO NPs, the XRD patterns revealed peaks characteristic of the hexagonal wurtzite structure of ZnO with space group P63mc (186). The samples from the polar extract exhibited strong diffraction peaks at 2θ values of 31.8°, 34.5°, 36.3°, 47.6°, 56.7°, 63.0°, and 68.1°, which correspond to the (100), (002), (101), (102), (110), (103), and (112) planes, respectively (JCPDS Card No. 75-0576). These results confirm the successful synthesis of ZnO NPs P with high crystallinity in the polar medium with a crystallite size of 28.2 nm. In contrast, the ZnO NPs A synthesized from the apolar extract showed slightly broader peaks with lower intensities, indicative of a smaller crystallite size of 22.4 nm. The apolar environment likely influenced the nucleation and growth of the ZnO NPs A, potentially leading to a higher degree of amorphization of smaller NPs [31].

The XRD patterns of Ag/Ag_2_O/ZnO NC P and Ag/Ag_2_O/ZnO NC A synthesized from polar and apolar extracts displayed a combination of diffraction peaks corresponding to Ag, Ag_2_O, and ZnO phases. In the polar-extract-derived Ag/Ag_2_O/ZnO NC P, the characteristic peaks of the cubic structures of Ag and Ag_2_O were visible with the hexagonal wurtzite structure of ZnO, with no significant shift in peak positions, indicating the co-existence of separate Ag, Ag_2_O, and ZnO phases within the NC. The crystallite size was estimated to be around 31 nm. For the Ag/Ag_2_O/ZnO A from the apolar extract, the XRD patterns also showed the presence of both Ag and ZnO phases, but with broader peaks and lower intensities, particularly for the ZnO-related peaks with a crystallite size of about 30 nm. This suggests that in the apolar phase, the ZnO NPs within the NC may be smaller or less crystalline, potentially due to interactions with the lipid components during synthesis. The presence of both Ag and ZnO phases confirms the successful formation of the nanocomposites in both environments, although the nature of the extract used influenced the structural properties. The presence of Ag_2_CO_3_ (JCPDS Card No. 70-2184) in the Ag/Ag_2_O/ZnO NC, as indicated by characteristic peaks in the XRD analysis, can be attributed to the interaction between silver ions and carbonate species originating from the extract used in the synthesis. The carbonate species can coordinate Ag^+^, enhancing their stabilization and facilitating their reaction to form Ag_2_CO_3_ through electrostatic attraction [32]. The presence of ZnO increases the availability of reactive sites for carbonate adsorption and improves the reaction kinetics, promoting the reduction of silver ions and stabilizing the intermediate phases during synthesis. In contrast, the formation of Ag_2_CO_3_ is less efficient in isolated Ag NPs or ZnO NPs due to the lack of sufficient reactive sites and interactions, underscoring the crucial role of ZnO in the NC structure.

The functionalization by the extract was studied using FTIR analysis (Figure 2B). The FTIR spectra of Ag NPs Sigma revealed no functional groups, while the Ag/Ag_2_O NPs synthesized from polar and apolar extracts revealed characteristic absorption bands associated with various functional groups on the NP surfaces. Both spectra showed a peak near 2976 cm^−1^, corresponding to C-H stretching vibrations from the aliphatic chains, indicating that lipid molecules in the apolar extract played a significant role in stabilizing the Ag/Ag_2_O NPs [33]. A band near 1628 cm^−1^ was observed, corresponding to the C=O stretching vibration of amides, suggesting interactions between silver and proteinaceous components in the extract [33]. The peaks at 1496 cm^−1^ and 1186 cm^−1^ can be attributed to the C=C stretching vibrations of aromatic rings and C-O stretching vibrations [34,35], respectively, likely due to the adsorption of organic molecules from the extract during synthesis.

In the FTIR spectra of the ZnO NPs P, the peaks at 1382 cm^−1^, 1176 cm^−1^, and 960 cm^−1^, are indicative of C-O stretching vibrations from carbonate groups, C-O-C bending, and Zn-O stretching [36,37,38,39], respectively, suggesting that organic capping agents and residual compounds from the synthesis process are present on the surface of the ZnO nanoparticles, along with characteristic Zn-O bonds. In ZnO NPs A from the apolar extract, the spectra also showed absorption bands around 2976 cm^−1^, consistent with the presence of lipid molecules, further supporting the role of the apolar medium in capping and stabilizing the ZnO NPs [33]. The FTIR spectra of the Ag/Ag_2_O/ZnO NC from the polar and apolar extracts combined features of both Ag NPs and ZnO NPs. In nanoparticles synthesized in the apolar phase, lipid-related peaks were observed, highlighting the role of lipids as reducing agents that facilitate the formation of nanoparticles while also creating a hydrophobic barrier that prevents aggregation. Conversely, nanoparticles synthesized in the polar phase interacted with polar molecules, such as proteins, carbohydrates, and other organic compounds, which also act as reducing and capping agents. These agents influence nanoparticle morphology and reactivity, potentially reducing cytotoxicity due to their protective layers. These findings align with the existing literature demonstrating that microalgae-derived biomolecules serve as natural capping agents, minimize nanoparticle agglomeration, and control metal ion release, thus mitigating oxidative stress [6]. The results indicate the presence of both Zn-O and organic capping agents on the NC surfaces, suggesting a successful integration of Ag, Ag_2_O, and ZnO components in the polar and apolar phases.

The differences between the obtained structures can be understood by following the synthesis process. Upon introducing AgNO_3_ or ZnSO_4_ into the diluted polar phase obtained from the Folch extraction, significant ion dissolution was observed due to the aqueous nature of the medium. The polar phase, rich in water and hydrophilic compounds, effectively facilitated the dissociation of the metal salts into their respective ions. The metal cations, once dissolved, likely interacted with various biomolecules present in the polar phase, such as proteins or small polar metabolites, forming metal complexes through coordination with functional groups like carboxyl, hydroxyl, or amino groups [40]. The controlled heating of the solution to 50 °C enhanced the ion mobility, promoting more effective interactions. After 15 min of reaction time, the gradual addition of NaOH raised the pH to approximately 8, inducing the precipitation of metal hydroxides. For silver, this process likely led to the formation of silver and silver oxide, which could be further reduced under the given conditions, forming Ag/Ag_2_O NPs. In the case of zinc, Zn(OH)_2_ precipitated and transformed after calcination into ZnO NPs [41]. The resulting nanoparticles in the polar phase are expected to be stabilized by surrounding biomolecules, which could act as capping agents, preventing agglomeration and influencing nanoparticle size and shape [41].

Furthermore, adding AgNO_3_ or ZnSO_4_ to the diluted apolar phase can lead to different interactions due to the hydrophobic nature of this phase. The apolar phase, primarily consisting of lipids and other hydrophobic molecules, provides a less favorable environment for metal ion dissolution. However, solubilization occurred, likely facilitated by water or emulsified polar compounds forming micelles or interacting directly with lipid molecules, particularly polar head groups of phospholipids. As the temperature was raised to 50 °C, the solubilized metal ions began interacting more actively with the lipid components, possibly forming coordination complexes stabilized by the apolar environment. The subsequent addition of NaOH led to a pH increase, promoting the precipitation of Ag/Ag_2_O NPs or ZnO NPs, similar to the reactions in the polar phase. However, in the apolar phase, the presence of lipids likely played a critical role in shaping the morphology of the nanoparticles. The lipids may have acted as capping agents by stabilizing the NPs and preventing aggregation [1].

Following the formation of Ag/Ag_2_O NPs, synthesized ZnO NPs, which had undergone calcination, were introduced into the reaction mixture. The addition of calcined ZnO NPs introduced a heterogeneous component into the system, allowing them to interact with the Ag/Ag_2_O NPs through physical adsorption or potential chemical interactions, forming NC structures. The calcination process ensured that the ZnO NPs were crystalline, thus providing a stable matrix. The hydrophobic environment of the apolar phase would have affected the interaction between the Ag/Ag_2_O NPs and the ZnO NPs. Lipid molecules might have adsorbed onto the surface of the ZnO NPs, creating a barrier that influenced how the ZnO NPs interacted with the Ag/Ag_2_O NPs. The formation of Ag/Ag_2_O/ZnO NC in this phase could be driven by physical proximity and possible surface interactions. However, the hydrophobic environment might limit the extent of direct chemical bonding between the nanoparticles.

The addition of calcined ZnO NPs after the synthesis of Ag/Ag_2_O NPs in both phases introduces a new dimension to the synthesis process, potentially leading to hybrid nanomaterials with enhanced or novel functionalities. The interplay between the different phases, metal ions, and nanoparticles could be further explored to optimize the properties of these composite materials for specific applications [42]. The different chemical environments of the polar and apolar phases significantly influenced the formation and characteristics of the nanoparticles. In the polar phase, the aqueous environment can promote efficient ion dissolution and interaction with biomolecules, forming nanoparticles that are potentially smaller and more uniform due to the stabilizing effects of hydrophilic capping agents. The ability of the polar phase to support efficient metal ion dissolution and complexation with biomolecules contrasts with the more restricted but lipid-mediated stabilization observed in the apolar phase. Extracts of varying polarity have previously been tested for the synthesis of silver nanoparticles for catalytic and antimicrobial applications, revealing their influence on morphology and size [43,44,45], and demonstrating the potential for a single species to yield diverse nanomaterials. Even though the Folch extraction method is well known, it has not previously been utilized for preparing extracts for nanoparticle synthesis.

Our findings provide valuable insights into the design of nanoparticle synthesis processes, particularly in terms of utilizing different solvent environments to tailor nanoparticle properties.

**Figure 3:**
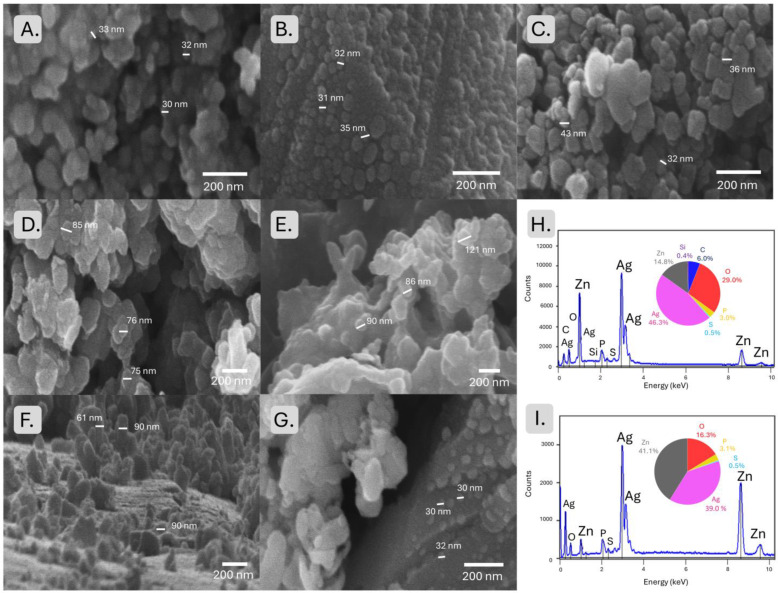
Microscopic analysis: (**A**) SEM of Ag NPs Sigma, (**B**) SEM of Ag/Ag_2_O NPs A, (**C**) SEM of Ag/Ag_2_O NPs P, (**D**) SEM of ZnO NPs A, (**E**) SEM of ZnO NPs P, (**F**) SEM of Ag/Ag_2_O/ZnO NC A, (**G**) SEM of Ag/Ag_2_O/ZnO NC P, (**H**) EDX of Ag/Ag_2_O/ZnO NC A, (**I**) EDX of Ag/Ag_2_O/ZnO NC P.

The morphology of the materials was analyzed by Scanning Electron Microscopy (SEM) with Energy-Dispersive X-ray Spectroscopy (EDX) (Figure 3). The SEM images of Ag NPs Sigma reveal uniformly dispersed nanoparticles with a relatively spherical shape, indicating homogeneous NP formation. The SEM images of the Ag/Ag_2_O NPs P synthesized from the polar extract revealed relatively uniform and spherical particles with an average size ranging from around 30 to 40 nm (Table 1). The particles appeared well dispersed, with minimal agglomeration, likely due to the stabilization provided by polar biomolecules acting as capping agents [46]. The surface morphology of the NPs suggests effective control over the growth process, facilitated by the aqueous medium of the polar phase. In contrast, the Ag/Ag_2_O NPs A synthesized from the apolar extract exhibited similar particle sizes varying between 28 and 35 nm. The SEM images show that the nanoparticles tended to form clusters, possibly due to the reduced steric stabilization in the apolar medium. The presence of lipid molecules in the apolar phase may have led to larger and less uniform nanoparticles forming, with some particles displaying irregular shapes and surface roughness.

The SEM analysis of the ZnO NPs P synthesized from the polar extract revealed that the ZnO NPs P had a predominantly hexagonal shape, typical of wurtzite ZnO structures, with particle sizes around 90 nm. The nanoparticles were well dispersed, suggesting that the polar medium provided sufficient stabilization during the synthesis process. The relatively uniform morphology and size distribution indicate a controlled nucleation and growth process facilitated by the aqueous environment [46]. However, the ZnO NPs A synthesized from the apolar extract displayed a more irregular morphology. SEM images showed a mixture of spherical and rod-like particles, with sizes ranging from 75 to 85 nm. The observed particle shape and size variability suggests that the apolar environment influenced the nucleation and growth processes differently than the polar phase. Lipids likely affected the crystallization and aggregation behavior, forming ZnO NPs A with more diverse morphologies.

SEM images of Ag/Ag_2_O/ZnO NC P synthesized from the polar extract revealed a complex morphology, with Ag/Ag_2_O NPs P appearing as smaller, spherical particles of around 30 nm distributed over the surface of larger ZnO NPs P. The Ag/Ag_2_O/ZnO NC P exhibited a relatively uniform distribution of Ag/Ag_2_O NP and ZnO NP components with minimal aggregation. The polar environment seemed to support the formation of well-defined nanocomposites; the Ag/Ag_2_O NPs P were effectively anchored onto the ZnO NP P surfaces, potentially enhancing their functional properties. For the Ag/Ag_2_O/ZnO NC A synthesized from the apolar extract, the SEM analysis showed more aggregated and heterogeneous structures around 60 to 90 nm. The NPs were observed to cluster together, with ZnO NPs A likely forming the core and Ag/Ag_2_O NPs A appearing as smaller particles attached to or embedded within the clusters. The apolar medium likely led to less efficient stabilization, resulting in larger agglomerates and more irregular composite structures. The lipid components in the apolar phase may have influenced the particle–particle interactions [47], leading to less distinct separation between the Ag, Ag_2_O, and ZnO phases.

EDX analysis was performed on the Ag/Ag_2_O/ZnO NC to confirm the elemental composition and distribution of Ag and Zn within the synthesized materials. The EDX spectra of the Ag/Ag_2_O/ZnO NC P synthesized from the polar extract showed prominent peaks corresponding to Ag and Zn, along with O, likely associated with the ZnO and Ag_2_O phases, with P and S from the extracts. The quantitative analysis revealed a homogeneous distribution of Ag and Zn elements across the composite, suggesting that Ag/Ag_2_O NPs were uniformly dispersed on the ZnO surface. In the case of the Ag/Ag_2_O/ZnO NC A synthesized from the apolar extract, the EDX spectra also confirmed the presence of Ag, Zn, and O, with C, P, and S from the extract. However, the elemental mapping revealed a less uniform distribution of Ag and Zn, with regions of higher Ag concentration indicating localized clusters or agglomerates of Ag/Ag_2_O NPs A. The apolar medium seemed to lead to more heterogeneous composites, with some areas showing higher ZnO content and others being richer in Ag. Despite this, the EDX results confirmed the successful synthesis of Ag/Ag_2_O/ZnO NC, albeit with a more variable distribution of the constituent elements.

The SEM and EDX analyses provided valuable insights into the morphological and compositional differences between the nanomaterials synthesized from the polar and apolar extracts. The SEM images indicated that the polar extract favored the formation of smaller, more uniform nanoparticles with better dispersion and well-defined composite structures. In contrast, the apolar extract led to larger, more heterogeneous nanoparticles with increased aggregation, particularly in the Ag/Ag_2_O/ZnO NC A. The EDX analysis further corroborated these findings, revealing more homogeneous elemental distributions in the polar-derived samples compared to the apolar-derived ones. These observations highlight the critical role of the synthesis environment in determining the final morphology and composition of nanoparticles and nanocomposites. The choice between polar and apolar phases can be strategically used to tailor the properties of the resulting nanomaterials for specific applications, depending on the desired particle size, morphology, and compositional uniformity.

### 2.2. Cytotoxicity of the Produced Nanoparticles

Ag/Ag_2_O NPs and ZnO NPs display diverse cytotoxic profiles when interacting with PC12 cells, influenced by their size, crystallinity, phase composition, surface morphology, and their ability to generate reactive oxygen species (ROS). A detailed assessment reveals notable differences among the Ag NPs, ZnO NPs, the commercially obtained Ag Sigma NPs, and the Ag/Ag_2_O/ZnO NCs.

#### 2.2.1. MTT Assay

The MTT assay revealed that Ag NPs, particularly Ag/Ag_2_O NPs A with an average size of 30.7 nm, exhibited significant cytotoxic effects on PC12 cells. As shown in Table 2, a low LD_50_ was detected as early as 12 h post-exposure, underscoring the rapid onset and strong potency of the toxicity.

Although smaller nanoparticles generally have a higher surface-area-to-volume ratio, the cytotoxicity of Ag/Ag_2_O NPs A appears to be more influenced by their surface reactivity and surface characteristics rather than size alone. These surface features likely promote interactions with intracellular components, leading to oxidative stress and cytotoxic effects [20]. This is consistent with findings showing that surface properties, such as the release of Ag^+^ ions and the interaction with biological molecules, are primary determinants of cytotoxicity rather than nanoparticle size. In fact, according to Powers et al., the cytotoxicity of Ag NPs arises not only from the release of Ag^+^ ions, but also from their surface characteristics, which play a crucial role in their interaction with cellular components [27]. Ag NPs disrupt mitochondrial function, leading to an increase in reactive oxygen species (ROS) production and the subsequent induction of oxidative stress. The disruption affects the electron transport chain, impairing ATP synthesis and promoting mitochondrial dysfunction [27]. The surface reactivity of Ag NPs enhances these interactions, which in turn lead to the damage of key cellular components such as proteins, lipids, and DNA, ultimately resulting in cell death through apoptosis or necrosis. Additionally, Sambale et al. highlighted that the cytotoxic potential of Ag NPs is strongly influenced by surface reactivity rather than size alone, underscoring the role of surface interactions in promoting oxidative damage and cell death [20,26].

The polarity of the synthesis medium also plays a crucial role in shaping the surface chemistry of Ag NPs and ZnO NPs, significantly influencing their cytotoxicity. For instance, ZnO NPs synthesized in polar environments (ZnO NPs P) exhibit higher crystallinity and lower LD_50_ values compared to those produced in apolar settings, indicating increased toxicity. Polar functional groups, such as hydroxyl (-OH) and carboxyl (-COOH), enhance the interaction of ZnO NPs P with cellular membranes, facilitating their uptake and promoting ROS generation. Elevated ROS levels, in turn, induce oxidative stress within the cells, damaging essential macromolecules such as proteins, lipids, and DNA [17,29]. The functional groups formed during polar synthesis improve membrane interactions, increasing nanoparticle internalization and amplifying oxidative damage. Furthermore, the enhanced crystalline structure of ZnO NPs, promoted by polar conditions, likely boosts their catalytic activity, leading to higher ROS production and intensified cytotoxic effects. ZnO NPs synthesized in polar media also demonstrate a higher tendency for ROS generation, resulting in oxidative stress that damages key cellular components, ultimately triggering apoptosis [17].

In contrast, ZnO NPs synthesized in apolar media (ZnO NPs A) exhibited reduced cytotoxicity, with higher LD_50_ values and a more amorphous structure. The surface capping derived from apolar media likely forms a hydrophobic barrier around the ZnO NPs, limiting their ability to generate ROS and interact with cellular membranes. The capping reduces Zn^2+^ ion release and hinders cellular uptake, resulting in diminished cytotoxicity. Previous studies have shown that surface modifications such as capping can play a crucial role in reducing nanoparticle cytotoxicity by limiting oxidative stress and ion release [10,28]. The reduced cellular uptake of apolar-synthesized ZnO NPs likely explains their lower ability to induce oxidative stress and the corresponding higher LD_50_ values observed in PC12 cells.

Similarly, the polarity of the synthesis environment plays a crucial role in influencing the cytotoxicity of Ag NPs. Indeed, when the latter are synthesized without stabilizing surface groups, such as in certain apolar environments, they exhibit greater cytotoxicity compared to their polar counterparts. The increased toxicity is likely due to the lack of surface stabilization, which leads to a more rapid release of Ag^+^ ions. Without protective surface capping, Ag^+^ ions are more freely dissociated, allowing them to aggressively interact with cellular components, inducing oxidative stress. The rapid release of Ag^+^ ions exacerbates their impact on mitochondrial function, causing increased ROS production and ultimately leading to cell death via apoptosis [19,27].

In contrast, Ag NPs synthesized in polar environments exhibit greater stability and reduced cytotoxicity. The presence of polar functional groups (e.g., amino acids and hydrophilic residues) on the surface of these Ag NPs forms a stabilizing layer, which reduces the rate of Ag^+^ ion release and mitigates their cytotoxic effects. By slowing down Ag^+^ ion release, polar-synthesized Ag NPs lower their interaction with cellular components, leading to less oxidative stress and reduced cytotoxicity [19]. The observed results support findings showing that the synthesis environment significantly affects nanoparticle reactivity and interaction with biological systems [10].

Ag/Ag_2_O/ZnO NCs demonstrated a hybrid cytotoxic profile, combining characteristics of both Ag and ZnO NPs. The polar-synthesized Ag/Ag_2_O/ZnO NC P exhibited a more crystalline structure and greater cytotoxicity than its apolar counterpart, with LD_50_ values decreasing over time (from 1.30 µg/mL at 12 h to 1.92 µg/mL at 48 h). The synergistic interaction between Ag and ZnO components likely amplifies toxic effects via surface reactivity and ROS generation, which is more pronounced in the polar medium due to the well-defined crystalline structures [17,20]. Conversely, apolar-synthesized Ag/Ag_2_O/ZnO NC A showed lower cytotoxicity, with relatively stable LD_50_ values over time. The lipid capping and lower crystallinity reduced reactivity and ROS production, limiting oxidative damage [10,20]. The amorphous structure of the synthesized NCs hinders their ability to generate ROS, thus resulting in reduced cytotoxicity compared to their polar-synthesized counterparts.

The polarity of the synthesis environment and the presence of functional groups on the nanoparticle surface significantly influence the cytotoxicity of ZnO and Ag NPs. Nanoparticles synthesized in polar environments generally exhibit higher crystallinity, increased ROS production, and greater cytotoxicity, whereas those synthesized in apolar environments show reduced cytotoxicity due to lipid capping and lower surface reactivity. Ag/Ag_2_O NPs exhibit comparable cytotoxicity from both apolar and polar extracts, while the ZnO NPs from the apolar extract demonstrate twice the toxicity of their polar counterparts. All these nanoparticles have a crystallite size within 20–30 nm, with smaller Ag/Ag_2_O NPs P than Ag/Ag_2_O NPs A and smaller ZnO NPs A than ZnO NPs P. However, the overall toxicity of Ag/Ag_2_O NPs is approximately 20 times higher than that of ZnO NPs, suggesting that size is likely not the determining factor in cytotoxicity. Interestingly, when Ag/Ag_2_O is deposited onto ZnO, there is a slight enhancement in cytotoxicity, which can be attributed to the presence of silver enhancing the ROS generation while simultaneously improving interaction with cellular components. Additionally, Ag/Ag_2_O NPs may facilitate increased uptake by cells, thus amplifying the overall cytotoxic effects. This combination leverages the distinct mechanisms of action from both Ag/Ag_2_O NPs and ZnO NPs, leading to enhanced efficacy in inducing cytotoxicity. The understanding of these relationships underscores the critical importance of nanoparticle surface chemistry in designing safer and more effective nanomaterials for therapeutic applications, particularly in cancer therapy. By tailoring the synthesis environment, cytotoxicity can be controlled and biological interactions optimized, paving the way for more targeted and efficient treatments.

To explore the selective effects of nanoparticles on proliferative versus differentiated PC12 cells, the MTT assay was performed at 24 h using the same nanoparticle concentrations as previously described. The analysis of LD_50_ values for each nanoparticle type in both cell phenotypes provided critical insights into their distinct responses. Using the ‘Comparison of Fits’ test, we identified significant differences between the dose–response curves (*p* < 0.0001), and the F-test confirmed the necessity of the distinct curves for each dataset (Figures S1–S7). These findings demonstrate how tailoring nanoparticle synthesis can influence cytotoxicity and highlight the variability in toxicity profiles across nanoparticle types and cell phenotypes. In fact, differentiated PC12 cells demonstrated greater resilience to ZnO NPs, with LD_50_ values exceeding the maximum tested concentration of 120 μg/mL (Figures S1–S7). In contrast, nanoparticles like the Ag/Ag_2_O NPs exhibited similar toxicity across both cell types (Table 3). These results underscore the importance of tailoring nanoparticle properties to minimize toxicity and optimize biological interactions while considering the differential sensitivity of cells based on their state of differentiation. In contrast, Ag/Ag_2_O NPs and Ag/Ag_2_O/ZnO nanocomposites demonstrated comparable toxicity in both cell types. For example, Ag/Ag_2_O NPs synthesized in apolar environments exhibited LD_50_ values of 0.89 μg/mL in non-differentiated cells and 0.81 μg/mL in differentiated cells, indicating a lack of selectivity and high intrinsic toxicity. Similarly, the Ag/Ag_2_O/ZnO nanocomposites showed minimal variation between the two cell states (e.g., 1.27 μg/mL vs. 1.37 μg/mL for apolar synthesis).

These findings suggest a dual mechanism: differentiated neuronal-like cells are more resistant to ZnO due to their specialized structure and function, while nanoparticles like Ag/Ag_2_O show uniform toxicity in both cell types, likely bypassing the defenses of differentiated cells through mechanisms like oxidative stress or membrane damage. Non-differentiated PC12 cells, with higher proliferative activity, are more vulnerable to nanoparticle-induced damage, whereas the resilience of differentiated cells may be linked to changes in metabolism, membrane composition, or reduced proliferation, although further studies are needed to confirm this. The variability in toxicity profiles, reflected by distinct LD_50_ values and dose–response curves, highlights the need to tailor nanoparticle synthesis to reduce toxicity and enhance biological compatibility. Synthesis conditions, such as the polarity of the environment, influence nanoparticle properties like crystallinity, surface reactivity, and ion release, which affect their interactions with PC12 cells. Gaining an understanding of these factors through neurotoxicity studies is essential for developing safer and more effective nanomaterials for biomedical applications such as cancer therapy.

#### 2.2.2. Role of ROS Generation in Nanoparticle-Induced Cytotoxicity

**Figure 4:**
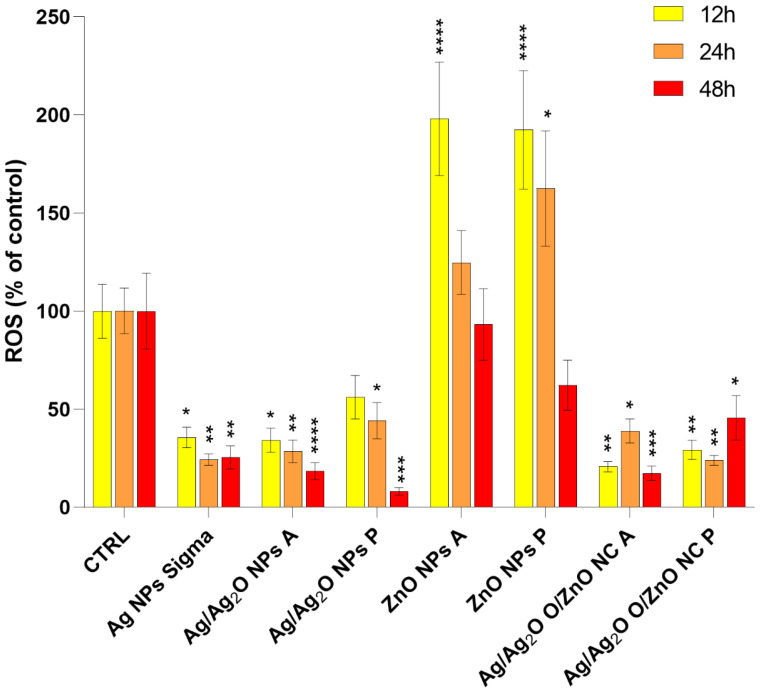
Reactive oxygen species (ROS) levels (% of control) generated by LD_50_ administration of different nanoparticles in PC12 cells after 12, 24, and 48 h of treatment. Mean differences were analyzed using two-way ANOVA followed by Dunnett’s correction for multiple comparisons (*n* = 12). Statistical significance was denoted as follows: ns for *p*-value > 0.05; * for *p*-value < 0.05; ** for *p*-value < 0.01; *** for *p*-value < 0.001; and **** for *p*-value < 0.0001.

ROS generation plays a critical role in nanoparticle-induced cytotoxicity, particularly in the case of ZnO NPs and Ag/Ag_2_O/ZnO NCs. The correlation between LD_50_ values and the ability to generate ROS indicates that oxidative stress is a key factor driving the cytotoxicity observed, especially for ZnO NPs synthesized in polar environments (ZnO NPs P) (Figure 4). The latter exhibited higher crystallinity, enhancing their catalytic activity and facilitating ROS generation. The crystalline structure of ZnO NPs P promotes efficient electron transfer, thereby catalyzing the production of ROS, which in turn leads to the oxidative damage of key cellular macromolecules such as proteins, lipids, and DNA, ultimately contributing to cell death [48]. The progressive increase in ROS levels observed with extended exposure to ZnO NPs P correlates with steadily decreasing LD_50_ values, indicating that oxidative stress accumulates over time, contributing to the higher cytotoxicity of these nanoparticles [49]. In contrast, ZnO NPs synthesized in apolar environments (ZnO NPs A) showed significantly reduced ROS generation. Likely, the lipid capping surrounding these nanoparticles acted as a protective barrier, limiting direct interaction with intracellular components and reducing surface reactivity. The reduction in ROS generation is consistent with the higher LD_50_ values and lower cytotoxicity observed for ZnO NPs A [49].

Regarding the Ag/Ag_2_O/ZnO NCs, their behavior concerning ROS generation is more complex due to the synergistic interaction between the Ag, Ag_2_O, and ZnO components, which amplifies their cytotoxicity. NCs synthesized in polar environments (NC P) exhibited more ordered crystalline structures and greater ROS-generating capacity compared to their apolar counterparts (NCs A). In NCs P, the synergy between Ag, Ag_2_O, and ZnO enhances ROS production and amplifies toxic effects. The well-defined crystalline structure and increased surface reactivity of polar-synthesized NCs facilitate more efficient ROS generation, resulting in significant oxidative stress and cell death [50].

This accounts for the lower LD_50_ values observed for NC P, reflecting a faster accumulation of ROS and heightened toxicity. Conversely, NCs synthesized in apolar environments (NCs A) demonstrated reduced cytotoxicity, with more stable LD_50_ values over time. The amorphous structure and lipid capping of these nanocomposites serve as a protective layer, limiting their interaction with intracellular components and reducing ROS production. This protective mechanism explains the lower oxidative stress and reduced toxicity observed in the NC A, as lipid capping not only diminishes surface reactivity but also prevents ROS accumulation, thereby reducing cellular damage [51].

Unlike ZnO NPs and NCs, Ag NPs generated lower levels of ROS despite their significant cytotoxicity, suggesting that their primary mechanism of toxicity is not ROS-mediated, but rather driven by the release of Ag^+^ ions. These ions interfere with critical cellular processes by binding to thiol (-SH) groups in intracellular proteins, leading to mitochondrial dysfunction and indirect oxidative stress. The rapid release of Ag^+^ ions from Ag NPs synthesized in apolar environments likely explains the high cytotoxicity observed, as the lack of surface stabilization facilitates more aggressive ion dissociation [52]. Conversely, Ag NPs synthesized in polar environments benefit from more stabilized surfaces, slowing the release of Ag^+^ ions and thereby reducing their toxic effects [52]. Thus, surface chemistry and the synthesis environment play a critical role in modulating the toxicity mechanisms of these nanoparticles. While ROS production is central to the cytotoxicity of ZnO NPs and NCs, Ag NPs exert their toxic effects primarily through an ion-mediated mechanism, highlighting the diversity in the pathways of toxicity among different nanoparticle types.

#### 2.2.3. Bioenergetic Function

**Figure 5:**
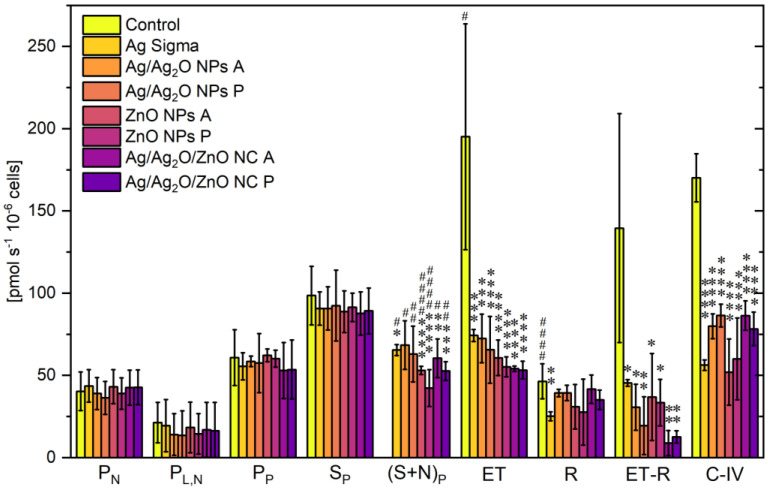
Mitochondrial respiration rates for undifferentiated PC12 cells (0.5 × 10^6^ cells mL^−1^): intact cell respiration with malate and pyruvate (P_N_); permeabilized cell respiration with malate and pyruvate (P_L,N_); OxPhos capacity with C-I substrates (malate and pyruvate, P_P_) and C-II substrates (succinate, S_P_); C-I and C-II OxPhos capacity after NP treatment ((S+N)_P_); maximum uncoupled respiration with CCCP (ET); uncoupled respiration with C-I inhibited by rotenone (R); contribution of C-I to uncoupled respiration (ET-R); uncoupled C-IV activity with ascorbate and TMPD (C-IV). The marks indicate statistical significance in terms of the difference from the corresponding respiratory state of the control group (*) or from the previous respiratory state of the same group (^#^), as follows: *, ^#^ (*p* < 0.05), **, ^##^ (*p* < 0.01), ***, and ****, ^####^ (*p* < 0.0001). Data are expressed as mean ± SD and were analyzed using one- and two-way ANOVA with Holm–Bonferroni correction for multiple comparisons (*n* = 8 for control group and *n* = 3 for each NP/NC group).

To evaluate the cytotoxic effects of NPs and NCs on metabolic activity, the bioenergetic function of undifferentiated PC12 cells was characterized through a respirometric assay. The activities of the main mitochondrial electron transport chain (ETC) complexes (C-I, C-II, and C-IV) were measured by the stimulation, inhibition, and uncoupling of the oxidative phosphorylation system (Figure 5). The intact (P_N_) or permeabilized (P_L,N_) cellular respiration rate was similar in all experimental groups. OxPhos (state 3) capacities were assessed, adding a saturated concentration of ADP in the presence of C-I (pyruvate, P_P_) and C-II (succinate S_P_) substrates, respectively. These cell respiration rates were similar in all cell groups. Then, the cell respiration rate declined with the titration of the NPs and NCs until they reached the LD_50_, as reported in Table 2. The respiration rate ((S+N)_P_) was reduced by 22% (Ag NPs Sigma), 17% (Ag/Ag_2_O NPs A), 19% (Ag/Ag_2_O NPs P), 40% (ZnO NPs A), 54% (ZnO NPs P), 26% (Ag/Ag_2_O/ZnO NC A), and 25% (Ag/Ag_2_O/ZnO NC P) of S_P_. The cell respiration rate was statistically reduced in all cell groups, indicating the significant influence on the ETC of all cell groups treated, excluding those with Ag/Ag_2_O NPs P and A. In the control cells, the uncoupled mitochondrial respiration rate (ET), which quantifies ETC capacity, was higher than the rate determined with coupled mitochondria, (S_P_) indicating that the phosphorylation system limits OxPhos. In contrast, the ET was similar to OxPhos (S_P_) for most of the cell groups treated with NPs or NCs, indicating that the phosphorylation system does not limit OxPhos. Indeed, ET was strongly reduced in all NP/NC groups (*p* < 0.001), suggesting an impairment of electron flow through complexes C-I and C-II.

The contribution of complex C-I to the ET was determined with the measure of cell respiration inhibiting C-I with rotenone (R) and calculating the cell respiration rate difference between ET and R for all cell groups investigated (ET-R). ET-R rates in all cells treated with NPs or NCs were significantly lower than those observed for the control group, indicating an important impairment of complex I. Specifically, the respiration rate (ET-R) was reduced by 67% (Ag NPs Sigma), 78% (Ag/Ag_2_O NPs A), 86% (Ag/Ag_2_O NPs P), 74% (ZnO NPs A), 76% (ZnO NPs P), 94% (Ag/Ag_2_O/ZnO NC A), and 91% (Ag/Ag_2_O/ZnO NC P) of the control group. Also, in cells treated with Ag NPs Sigma, the respiration rate with rotenone was significantly lower than that of the control group, suggesting an additional effect of Ag NPs on the ETC not related to C-I.

Furthermore, the C-IV activity was characterized by the difference between the respiratory rates with ascorbate and TMPD (AsTm) and the auto-oxidation chemical background with sodium azide (Azd), which are not reported in Figure 5. C-IV activity was significantly lower in all NP/NC groups (*p* < 0.0001) compared to the control group. For all cell groups treated with NPs, this respiration rate (C-IV) was reduced by 67% (Ag NPs Sigma), 53% (Ag/Ag_2_O NPs A), 49% (Ag/Ag_2_O NPs P), 69% (ZnO NPs A), 65% (ZnO NPs P), 49% (Ag/Ag_2_O/ZnO NC A), and 54% (Ag/Ag_2_O/ZnO NC P) of the control group.

In conclusion, the NCs caused an alteration in OxPhos (S_P_), although all NPs or NCs reduced ETC capacity (ET). Thus, the presence of ZnO appears to be a determinant causing mitochondrial dysfunction under a coupling state. It should be noted that the metabolic difference observed between the NP and NC treatments cannot be attributed to the initial cellular metabolic activity because the respiration rates of the intact (P_N_) and permeabilized (P_L,N_) cells were similar to the control group for all cells investigated.

NPs, such as Ag/Ag_2_O and ZnO, present toxic interaction at cellular and molecular levels in biological systems. The dysfunction of the mitochondrial complexes responsible for oxidative phosphorylation leads to an impairment in energy production [53].

**Figure 6:**
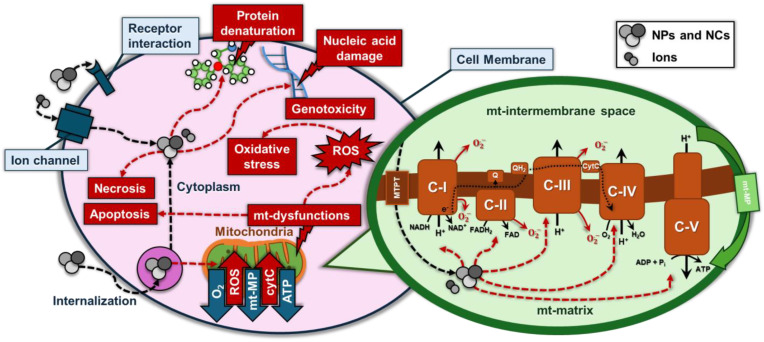
Schematic representation of the intake pathways of NP- or NC-treated cells, detailing their interactions at the cellular and mitochondrial levels. These NPs induce excessive ROS production, leading to oxidative stress and damage to proteins and DNA. In the mitochondria, they disrupt key complexes of the ETC, reducing ATP production and increasing ROS. This mitochondrial dysfunction, along with the loss of membrane potential, can ultimately trigger apoptosis and necrosis, leading to cell death. This figure is based on the internalization mechanisms proposed by Akter et al. [10].

Consistently, in our study, Ag/Ag_2_O- and ZnO-based NPs and NCs showed an interaction between their Ag^+^ and Zn^2+^ ion forms and complexes I and IV, reducing their activity and function (Figure 6). Our evidence cannot exclude an effect on mitochondrial membrane potential as well. Other studies have shown that Ag/Ag_2_O- or ZnO-based NPs induce ETC enzyme inhibition with ROS generation in various tissues, including the liver, brain, heart, muscle, and neuronal cells [54,55]. In addition, the magnitude of the cytotoxicity induced by Ag NPs is highly dependent on cell type and the energy demand on mitochondrial sources [56].

Cellular antioxidant defenses are overwhelmed in the presence of excessive ROS production and cause oxidative stress [57]. ETC dysfunction leads to excessive ROS production, thus contributing to oxidative stress. One of the key targets of ROS is mitochondrial DNA (mtDNA), leading to a cascade of events. mtDNA damage by oxidative stress impairs the replication and transcription of essential mitochondrial genes, further exacerbating mitochondrial dysfunction, leading to a decline in energy production. In addition to damaging mtDNA, ROS can modify and denature mitochondrial proteins, including components of respiratory complexes [57]. The protein denaturation impairs their functionality, further hindering ATP synthesis.

In summary, it is possible that the ion forms Ag^+^ and Zn^2+^ from NPs and NCs can alter mitochondrial function, with specific detrimental effects on complexes I and IV in undifferentiated PC12 cells. For ZnO NPs, cytotoxicity is mainly driven by ROS production, which is enhanced for nanoparticles synthesized in polar environments and leads to oxidative stress, damaging cellular macromolecules and causing cell death. In contrast, for Ag/Ag_2_O NPs, the primary cytotoxic mechanism is the release of Ag^+^ ions, especially from nanoparticles synthesized in apolar environments. The ions disrupt critical cellular functions, such as binding to thiol groups in proteins, leading to mitochondrial dysfunction and oxidative stress. Thus, while ROS generation is key for ZnO NP toxicity, Ag^+^ ion release plays a more significant role in Ag NP toxicity, underscoring the differing mechanisms of action between the two nanoparticle types. In addition to this effect, oxidative damage could contribute to the observed bioenergetic dysfunction. Considering the diverse functions of mitochondria in both physiological and pathological contexts, damaged mitochondria serve to balance survival and death and ultimately dictate cellular fate, which is why the effect of nanoparticle toxicity should be studied at various time points [58]. In addition, in previous studies, it was reported that ROS, ATP, mitochondrial membrane potential, tubular mitochondria, and the expression of Drp1 are relatively sensitive indicators of subcellular response to nanoparticles, with material type being a main influence, which is supported by our study [59]. Furthermore, this mitochondrial dysfunction, combined with increased oxidative stress, can initiate apoptotic pathways, leading to cell death.

## 3. Materials and Methods

### 3.1. Synthesis of ZnO Nanoparticles Using Extracts from Chlorella vulgaris Biomass

The microalga *Chlorella vulgaris* (CCALA 902) was cultivated in Bold’s Basal Medium (BBM) supplemented with 60 mM NaHCO_3_ to obtain biomass for further analysis. The culture was incubated under 12/12 light/dark-cycle 58W fluorescent lamps (Osram^®^, Milan, Italy) with a 70 μmol/m^2^/s^1^ photon flux. The cultures were maintained at room temperature with 200 rpm to ensure the uniform distribution of cells and nutrients. The microalgal cultures were allowed to grow for 14 days, with periodic optical density (OD) monitoring at 750 nm to assess growth. The cultures were harvested when they reached the exponential growth phase, indicated by an OD_750_ of approximately 0.6. After the cultivation period, the *C. vulgaris* biomass was harvested by centrifugation at 2000× *g* rpm for 10 min (Heraeus Megafuge 1.0R, Thermo Fisher Scientific, Milan, Italy) and dried at room temperature. The obtained biomass was subjected to the Folch extraction method [60]. Approximately 5 g of the sample was suspended in 100 mL of methanol and left in the fridge at 4 °C overnight. Afterward, the content was sonicated in an ultrasonic bath for 30 min to disrupt the cell membrane and release the cell content. Then, 200 mL of chloroform was added to the mixture and stirred at room temperature for 1 h at 300 rpm. The mixture was centrifuged at 4000× *g* rpm, 20 °C, for 10 min, and the biomass was discarded. The phase separation was induced by adding 60 mL of 0.88% KCl solution, and the solution was centrifuged again. After centrifugation, the upper aqueous phase (polar phase) was carefully removed using a Pasteur pipette, leaving the lower organic phase (apolar phase) containing the lipids. Both extracts were put under a rotary evaporator (Rotavapor R-210, Buchi, Milan, Italy) to remove the solvents. In the last stage, 150 mL of MilliQ H_2_O (Millipore^®^, Milan, Italy) was added to each phase, and the solutions were stored at 4 °C for further processing.

For the synthesis, the apolar and polar phases were diluted 1:10 with distilled water to create an optimal environment for nanoparticle formation, with a total volume of 300 mL for the Ag/Ag_2_O NPs and 600 mL for the ZnO NPs. The flasks were continuously stirred at 250 RPM and heated to 50 °C (IKA^®^ RH Digital Magnetic Stirrer, Milan, Italy). Once the temperature was stabilized, 0.1 M of the precursor salt (AgNO_3_, Carlo Erba, or ZnSO_4_ · 7 H_2_O, Merck, Milan, Italy) was added, and the mixture was allowed to react for 15 min. Subsequently, the pH of the reaction mixture was gradually increased by adding 1.25 M NaOH solution dropwise while maintaining constant stirring. The addition of NaOH was continued until the pH reached approximately 8, which is conducive to nanoparticle formation, to ensure the controlled nucleation and growth of the nanoparticles. The reaction was monitored by the gradual color change of the solution, with a yellowish-brown color for Ag/Ag_2_O NPs and a white color for ZnO NPs. The reaction continued for 90 min to ensure complete nanoparticle synthesis and maturation overnight at room temperature. The synthesized nanoparticles were then separated by centrifugation at 4000× *g* rpm for 15 min, washed with MiliQ H_2_O, and dried at 90 °C overnight. The ZnO NPs were subjected to calcination at 500 °C for 2 h. The Ag/Ag_2_O/ZnO NCs were synthesized using a similar methodology. A total volume of 150 mL with the same dilution ratio was stirred and heated, followed by the addition of 0.1 M AgNO_3_. After 15 min, the pH was raised to 8 by adding 1.25 M of NaOH, followed by 90 min of incubation. Then, 2 g of ZnO NPs were added to the flask, and the solution was maturated overnight at room temperature. Similarly, the product was centrifuged and dried at 90 °C for 12 h. The products were labeled according to the detected phases (Ag, Ag_2_O, or ZnO NPs—nanoparticles; NC—nanocomposite) and the extract was used (A—apolar; P—polar).

### 3.2. Characterization of Synthesized Nanoparticles

The crystalline structure and phase composition of the synthesized nanoparticles were determined using X-ray diffraction (XRD, Philips PW 1830, Amsterdam, The Netherlands). XRD patterns were obtained using a D8 Advance Bruker AXS equipped with a Cu Kα radiation source (λ = 1.5406 Å) operated at 40 kV and 30 mA. The samples were scanned over a 2θ range of 5° to 90° at a step size of 0.02°. The diffraction peaks were analyzed and compared with standard reference patterns in the International Centre for Diffraction Data (ICDD) and Crystallography Open Database (COD) to identify the phases present in the samples using Diffrac.Eva software v.6.1.0.4 (Bruker, Milan, Italy).

Fourier Transform Infrared (FTIR) spectroscopy was employed to identify the functional groups present on the surface of the nanoparticles. FTIR spectra were recorded using a FT/IR-6700, Jasco, Tokyo, Japan, equipped with a diamond attenuated total reflectance (ATR) accessory. Samples were prepared by directly placing the powdered nanoparticles onto the ATR crystal. Spectra were collected over the 4000–400 cm^−1^ range with a resolution of 4 cm^−1^ and averaged over 64 scans to improve the signal-to-noise ratio. The resulting spectra were analyzed to identify characteristic absorption bands corresponding to specific functional groups.

The morphology and elemental composition of the synthesized nanoparticles were examined using Scanning Electron Microscopy (SEM) coupled with Energy-Dispersive X-ray Spectroscopy (EDX). SEM images were acquired using a Hitachi S4000 FEG HRSEM (Hitachi Ltd., Tokyo, Japan) operating at an accelerating voltage of 20 kV. The samples were sputter-coated with a 2 nm layer of platinum and gold to enhance conductivity and reduce charging effects. The images were captured at various magnifications with the image acquisition software Quartz PCI, version 7.0, (Quartz Imaging Corporation, VAN, Canada) to analyze the size, shape, and surface morphology. For elemental analysis, EDX was performed on selected regions of the SEM images to determine the elemental composition and distribution within the nanoparticles. The EDX spectra were obtained with an attached UltraDry EDX Detector (Thermo Fisher Scientific, Madison, WI, USA) and analyzed using NSS3 software, version 3, (Thermo Fisher Scientific, Madison, WI, USA) to quantify the elemental composition, mainly focusing on the presence and ratio of elements such as Ag, Zn, O, C, etc.

### 3.3. Cell Culture Conditions

PC12 (CRL-1721) cells, obtained from the ATCC (American Type Culture Collection), were cultured in RPMI 1640 medium supplemented with 10% fetal bovine serum (FBS) and 1% penicillin–streptomycin, and incubated at 37 °C in a humidified atmosphere containing 5% CO_2_. Cells were routinely passaged upon reaching 80% confluence to ensure optimal growth conditions. For experimental use, cells were seeded onto plates pre-coated with poly-D-lysine to enhance cell adhesion and growth consistency. All experiments were performed using cells between passages 5 and 10 to maintain reproducibility and minimize phenotypic drift.

For differentiation, the medium was supplemented with 0.1% FBS and 100 ng/mL nerve growth factor (NGF) and refreshed every 48 h for 7 days. Cells between passages 5 and 10 were used to ensure consistent responses. This protocol promotes neuronal-like traits, including neurite outgrowth, branching, varicosities, and the expression of neuronal markers, while reducing the proliferative capacity of undifferentiated cells [61]. Evaluating both differentiated and undifferentiated cells is crucial to understanding nanoparticle-induced effects, as differentiation alters sensitivity through structural and functional adaptations [62].

### 3.4. Nanoparticle Cytotoxicity in PC12 Cells via MTT Assay

PC12 cells were seeded into 96-well plates at a density of 1 × 10^4^ cells per well/100 μL and incubated for 24 h to allow for cell adhesion. Following this, varying concentrations of nanoparticles (NPs) (0.1, 0.2, 0.3, 0.4, 0.5, 0.6, 0.9, 1.9, 3.8, 7.5, 15, 30, 60, and 120 μg/mL) were added to the wells to determine the IC50, while control wells received an equal volume of RPMI 1640 medium. At the end of the different time points of exposure (12, 24, and 48 h), cell viability was assessed using the MTT assay. A 20 μL aliquot of MTT solution (2.5 mg/mL) was added to each well and incubated for 4 h in the dark. After incubation, the supernatants were carefully removed, and 100 μL of DMSO was added to dissolve the formazan crystals. Absorbance at 570 nm was measured using a microplate reader (Victor X5 2030 Multilabel HTS Fluorescence, Perkin Elmer, Berlin, Germany).

The same protocol was performed for differentiated PC12 cells at the 24 h time point. These cells were differentiated directly within the multi-well plates by incubating them with RPMI-1640 medium supplemented with 0.1% FBS and 100 ng/mL NGF for 7 days prior to the addition of nanoparticles.

Cell viability was calculated as a percentage using the following formula: cell survival rate (%) = (OD_570_ of experimental group/OD_570_ of control group) × 100. All the experiments are expressed as mean % of the control ± standard deviation (SD) (*n* = 6).

### 3.5. Measurement of ROS Production in PC12 Cells

Reactive oxygen species (ROS) generation was quantified using the cell-permeable probe 2′,7′-dichlorodihydrofluorescein diacetate (DCFH-DA). Upon entering the cells, DCFH-DA is hydrolyzed by intracellular esterases to form the non-fluorescent compound 2′,7′-dichlorodihydrofluorescein (DCFH), which is rapidly oxidized to highly fluorescent 2′,7′-dichlorofluorescein (DCF) in the presence of ROS. A stock solution of DCFH-DA was prepared in DMSO at a concentration of 25 mM and stored at −20 °C. PC12 cells (1 × 10^4^/100 µL) were seeded into a 96-well microplate and incubated with 25 µM DCFH-DA for 30 min at 37 °C in the dark. The fluorescence of DCF was measured using a microplate reader (Perkin Elmer Victor X5 2030 Multilabel HTS Fluorescence) at room temperature with excitation and emission wavelengths of 485 nm and 530 nm, respectively. Background fluorescence was subtracted from the total fluorescence, and the results are expressed as a percentage of the positive control. All the experiments are expressed as mean % of the control ± SD (*n* = 12).

### 3.6. Measurement of Bioenergetic Function

Mitochondrial respiration rate was measured using a high-resolution respirometry system (Oroboros-O2k, Innsbruck, Austria). The following reagents used for respirometry were purchased from Sigma-Aldrich (St. Louis, MI, USA): malate (M), pyruvate (P), adenosine diphosphate (ADP), succinate (S), ascorbate (As), tetramethyl-p-phenylenediamine (TMPD), carbonyl cyanide 3-chlorophenylhydrazone (CCCP), rotenone (R), antimycin A (Ama), sodium azide (Azd), and digitonin (Dig). The mitochondrial respiration medium (MiR05) was prepared by mixing together ethylene glycol-bis(2-aminoethylether)-N,N,N′,N′-tetraacetic acid (EGTA, 0.5 mM), magnesium chloride hexahydrate (MgCl_2_·6H_2_O, 3 mM), lactobionic acid (K-lactobionate, 60 mM), taurine (20 mM), potassium dihydrogen phosphate (KH_2_PO_4_, 10 mM), HEPES (20 mM), D-sucrose (110 mM), and bovine serum albumin (BSA, 1 g/L, essentially fatty acid free). The pH of MiR05 was adjusted to 7.1 with KOH at 30 °C [63].

The PC12 sample was prepared in MiR05 and was added to the metabolic chamber to reach a final concentration of 0.5 × 10^6^ cells mL^−1^ in a total volume of 2 mL. The metabolic chamber temperature and stirring were set to 37 °C and 750 rpm, respectively. The polarographic oxygen sensors (POSs) were calibrated. DatLab 7.1 software (Oroboros Instruments, Innsbruck, Austria) was used for data processing and acquisition [64]. Respirometry was performed in a O_2_ concentration range of 75–195 μM. All rates were corrected for the non-mitochondrial O_2_ consumption rate obtained in the presence of antimycin A (C-III inhibitor) and are expressed in pmol s^−1^ 10^−6^ cells. Digitonin titration was performed to determine the concentration for optimal permeabilization in undifferentiated PC12 cells in the presence of rotenone (1.875 μM), succinate (10 mM), and ADP (2.5 mM). Oxidative phosphorylation (OxPhos) and electron transport chain (ETC) capacities were assessed in permeabilized cells by adding substrates, inhibitors, and uncouplers of complex I (C-I), complex II (C-II), and complex IV (C-IV), as previously described. The respirometry protocol followed the following sequence [65,66]: malate (3.2 mM), pyruvate (2.5 mM), digitonin (8 μg/mL), ADP (2.5 mM), succinate (10 mM), CCCP (0.5–12.5 μM), rotenone (1.875 μM), antimycin A (0.5 μM), ascorbate (2 mM), TMPD (0.5 mM), and sodium azide (200 mM). All values reference the final concentration within the metabolic chamber. NPs and NCs were titrated up to their LD_50_ (Table 2) after the saturation of OxPhos (S_P_) and before the uncoupling (ET).

### 3.7. Statistical Analysis

A dose–response curve was generated based on the percentage of cell viability at various concentrations of the treatment. Nonlinear regression analysis was performed to fit the data to a sigmoidal dose–response (variable slope) model. The LD_50_ value was calculated as the concentration at which 50% of the cells remained viable compared to the untreated controls. All data points represent the mean % of the control ± (SD) (*n* = 6). ROS levels are expressed as the mean % of the control ± SD (*n* = 12). Statistical comparisons were performed using a one-way ANOVA, followed by Dunnett’s post hoc test for multiple comparisons. Statistical significance is indicated as follows: ns (not significant, *p* > 0.05), * (*p* < 0.05), ** (*p* < 0.01), *** (*p* < 0.001), **** (*p* < 0.0001). All analyses were performed using GraphPad Prism 9.

Respiration rates are expressed as mean ± SD. Statistical significance was set at *p*-value < 0.05 and analyzed using OriginPro^®^ 2021 software (OriginLab Corporation, Northampton, MA, USA). One-way ANOVA was used to compare the control and other groups’ respiration rates before treatment, and two-way ANOVA with Holm–Bonferroni correction for multiple comparisons was used to compare the respiration rates between the control (*n* = 8) and NP/NC-treated (*n* = 3) groups in S_P_ and (S+N)_P_ states. The marks used indicate statistical significance in terms of the difference from the corresponding respiratory state of the control group (*) or from the previous respiratory state of the same group (^#^), as follows: *, ^#^ (*p* < 0.05), **, ^##^ (*p* < 0.01), ***, ^###^ (*p* < 0.001), and ****, ^####^ (*p* < 0.0001).

## 4. Conclusions

The green synthesis of Ag/Ag_2_O NPs, ZnO NPs, and Ag/Ag_2_O/ZnO NCs using polar and apolar extracts of *C. vulgaris* offers a sustainable and versatile method for producing nanoparticles with customizable properties. This method not only minimizes environmental impact, but also enables the fine-tuning of NP characteristics, including cytotoxicity, cellular interactions, and ROS generation capacity. The observed variability in cytotoxicity may be influenced by factors such as NP size and polarity. Cytotoxic modifications might be influenced by smaller nanoparticle sizes, particularly for Ag/Ag_2_O NPs synthesized with polar extracts, which experience an enhanced interaction with cellular components due to their increased surface-area-to-volume ratio. Although commercial Ag NPs exhibited even smaller crystallite sizes, their anticancer activity was not as effective, underscoring the importance of organic content in enhancing the therapeutic potential of nanoparticles. Additionally, nanoparticles produced in polar media tend to exhibit higher reactivity, leading to greater ROS generation and increased cytotoxicity, as seen in the ZnO NPs from the polar extracts. The presence of polar functional groups, such as hydroxyl and carboxyl, further enhances cellular uptake and ROS generation. Conversely, biocompatible modifications are associated with larger nanoparticle sizes found in apolar media, which reduce reactivity due to lipid capping. Lipid capping decreases the release of toxic ions and ROS generation, contributing to enhanced biocompatibility, as observed in ZnO and Ag/Ag_2_O/ZnO nanocomposites synthesized with apolar extracts.

Differentiated PC12 cells demonstrated greater resilience to the nanoparticles compared to their non-differentiated counterparts. This differential sensitivity highlights the critical influence of cellular type and state in determining responses to nanoparticles, particularly those with high ROS generation capacity. Conversely, non-differentiated cells, characterized by higher metabolic activity, are more susceptible to nanoparticle-induced damage.

The presence of bioactive compounds highlights the critical importance of precise NP design to maximize therapeutic efficacy while minimizing risk. Further investigation is required to fully understand the molecular pathways involved in these processes and their implications for the development of NP-based cancer therapies. Additionally, further research should explore the impact of the bioactive compounds in *C. vulgaris* extracts on the biocompatibility and biological interactions of NPs. Such studies could drive advances in the development of safer and more effective therapeutic and diagnostic strategies for targeting cancer cells.

## Figures and Tables

**Table 1:** Crystallite size, phase composition, and SEM morphology of Ag, ZnO nanoparticles, and Ag/Ag_2_O/ZnO nanocomposites synthesized in apolar (A) and polar (P) environments. SEM analysis reveals differences in nanoparticle shape and aggregation, while XRD analysis provides data on the crystalline structure and size.

Nanoparticle Type	Crystallite Size (nm)	Phase Composition	Morphology (SEM)
Ag NPs Sigma	14.4	Cubic Fm-3m (Ag)	Uniformly dispersed, spherical shape
Ag/Ag_2_O NPs A	30.7	Cubic Pn-3m (Ag) and Cubic Pn-3m (Ag_2_O)	Spherical particles, tend to form clusters
Ag/Ag_2_O NPs P	24.8	Cubic Pn-3m (Ag) and Cubic Pn-3m (Ag_2_O)	Relatively uniform, spherical particles with minimal agglomeration
ZnO NPs A	22.4	Hexagonal P63mc Wurtzite (ZnO)	Mixture of spherical and rod-like particles, diverse shapes
ZnO NPs P	28.2	Hexagonal P63mc Wurtzite (ZnO)	Predominantly hexagonal, well dispersed
Ag/Ag_2_O/ZnO NC A	30.0	Cubic Pn-3m (Ag), Cubic Pn-3m (Ag_2_O), and Hexagonal P63mc Wurtzite (ZnO)	Aggregated, heterogeneous structures, larger clusters
Ag/Ag_2_O/ZnO NC P	31.4	Cubic Pn-3m (Ag), Cubic Pn-3m (Ag_2_O), and Hexagonal P63mc Wurtzite (ZnO)	Smaller spherical particles anchored on larger ZnO surfaces

**Table 2:** Lethal-dose (LD_50_) values (µg/mL) of Ag/Ag_2_O, ZnO nanoparticles, and Ag/Ag_2_O/ZnO nanocomposites on PC12 cells at 12, 24, and 48 h. A and P indicate synthesis in apolar and polar environments, respectively.

NPs and NCs	12 h	24 h	48 h
Ag NPs Sigma	>120	>120	>120
Ag/Ag_2_O NPs A	0.59	0.89	0.72
Ag/Ag_2_O NPs P	0.75	0.88	0.92
ZnO NPs A	21.36	11.27	8.78
ZnO NPs P	39.21	21.32	10.53
Ag/Ag_2_O/ZnO NC A	1.72	1.27	1.49
Ag/Ag_2_O/ZnO NC P	1.30	1.30	1.92

**Table 3:** Lethal-dose (LD_50_) values (μg/mL) for Ag/Ag_2_O, ZnO nanoparticles and Ag/Ag_2_O/ZnO nanocomposites on non-differentiated (ND) and differentiated (D) PC12 cells after 24 h of exposure. Significant differences were observed between the dose–response curves (*p* < 0.0001), and the F-test confirmed the necessity of using distinct curves for each dataset.

LD_50_ (μg/mL) of Nanoparticles and Nanocomposites		
NPs and NCs	ND	D
Ag NPs Sigma	>120 *	>120 *
Ag/Ag_2_O NPs A	0.89	0.81
Ag/Ag_2_O NPs P	0.88	1.42
ZnO NPs A	11.27	>120 *
ZnO NPs P	21.32	>120 *
Ag/Ag_2_O O/ZnO NC A	1.27	1.37
Ag/Ag_2_O O/ZnO NC P	1.30	1.80

* 120 (μg/mL) is the maximum tested concentration.

## Data Availability

Data will be available upon request.

## Supplementary Materials

The following supporting information can be downloaded at: https://www.mdpi.com/article/10.3390/md22120549/s1, Figures S1–S7: Comparison of Fits analysis of dose-response curves.
