# Supplementary material for: Cytotoxic Effects of ZnO and Ag Nanoparticles Synthesized in Microalgae Extracts on PC12 Cells
Source: Mar Drugs. 2024 Dec 4;22(12):549. doi: 10.3390/md22120549 (PMC11677574; doi:10.3390/md22120549)
Supplement: Supplementary file 1 [file marinedrugs-22-00549-s001.zip › marinedrugs-3267302-supplementary.pdf]

Cytotoxic Effects of ZnO and Ag Nanoparticles Synthesized in Microalgae Extracts on PC12 Cells

Giacomo Fais [1,2,†], Agnieszka Sidorowicz [1,2,†], Giovanni Perra [1,2], Debora Dessì [3], Francesco Loy [4], Nicola Lai [1,2], Paolo Follesa [3], Roberto Orrù [1,2,*], Giacomo Cao [1,2,5] and Alessandro Concas [1,2,*]

[1] Interdepartmental Centre of Environmental Science and Engineering (CINSA), University of Cagliari, Via San Giorgio 12, 09124 Cagliari, Italy; giacomo.fais@unica.it (G.F.); sid.agnieszka@gmail.com (A.S.); giovanni.perra@unica.it (G.P.); nicola.lai@unica.it (N.L.); giacomo.cao@unica.it (G.C.)

[2] Department of Mechanical, Chemical and Materials Engineering, University of Cagliari, Via Marengo 2, 09123 Cagliari, Italy

[3] Department of Life and Environmental Sciences, University of Cagliari, Cittadella Universitaria di Monserrato, Monserrato, 09042 Cagliari, Italy; deboradessi95@gmail.com (D.D.); paolo.follesa@unica.it (P.F.)

[4] Department of Biomedical Sciences, Section of Cytomorphology, University of Cagliari, Cittadella Universitaria di Monserrato, Monserrato, 09042 Cagliari, Italy; floy@unica.it

[5] Center for Advanced Studies, Research and Development in Sardinia (CRS4), Loc. Piscina Manna, Building 1, 09050 Pula, Italy

* Correspondence: roberto.orru@unica.it (R.O.); alessandro.concas@unica.it (A.C.)

† These authors contributed equally to this work.

Ag NPs Sigma

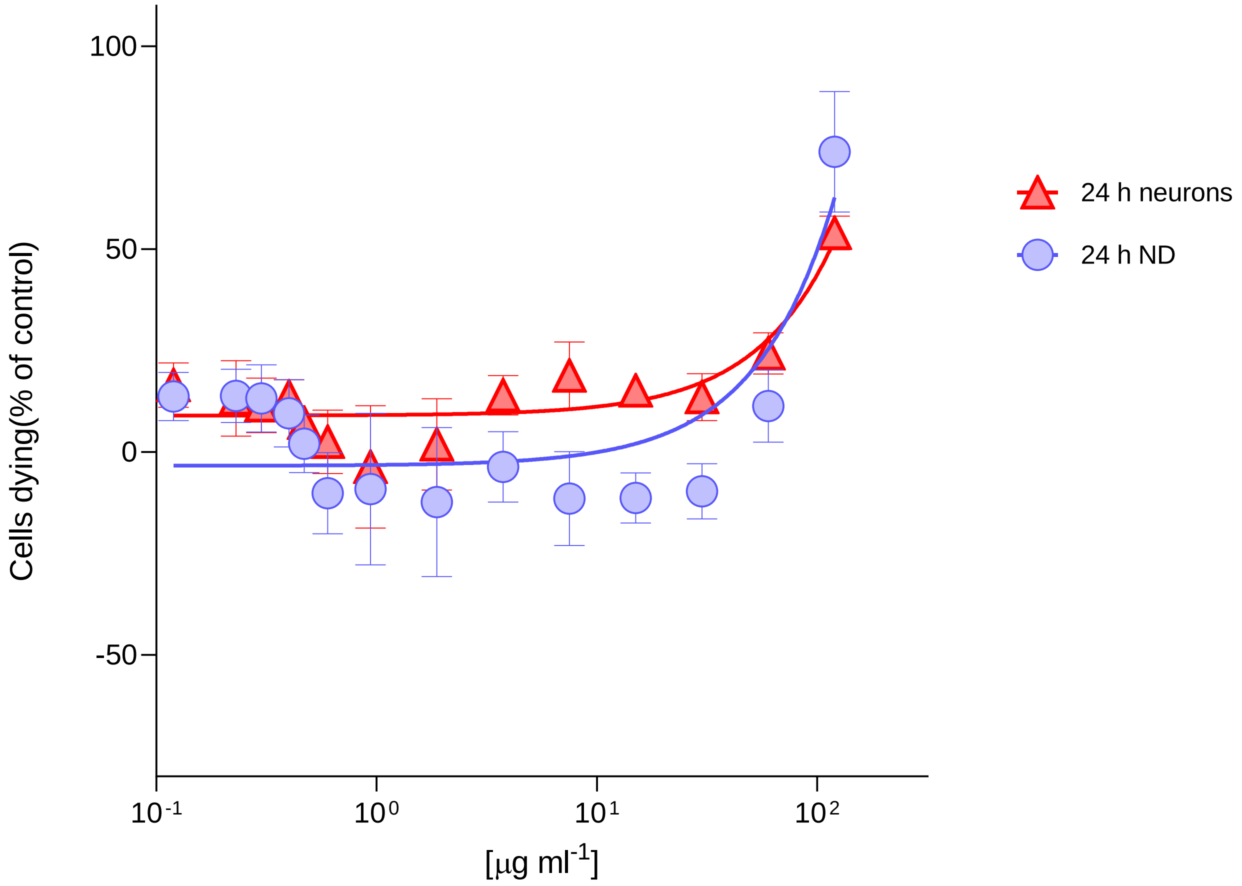

Figure S1. Comparison of Fits analysis demonstrates statistically significant differences among data sets ($P < 0.0001$), supporting the use of distinct curves for each set ($F(4, 160) = 11.80$).

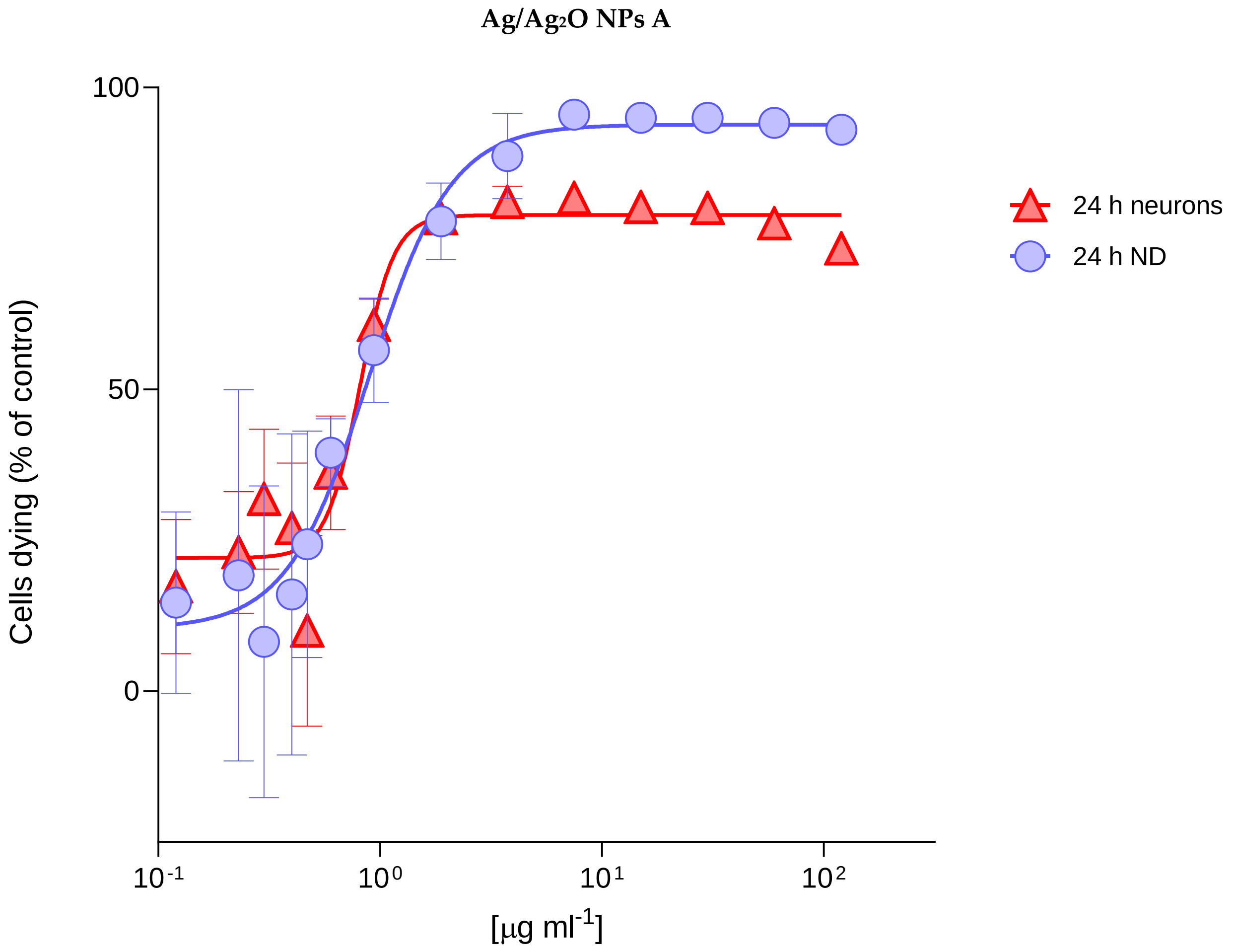

Figure S2. Comparison of Fits analysis indicates significant differences among data sets ($P < 0.0001$), favoring distinct curves for each set ($F(4, 160) = 8.09$).

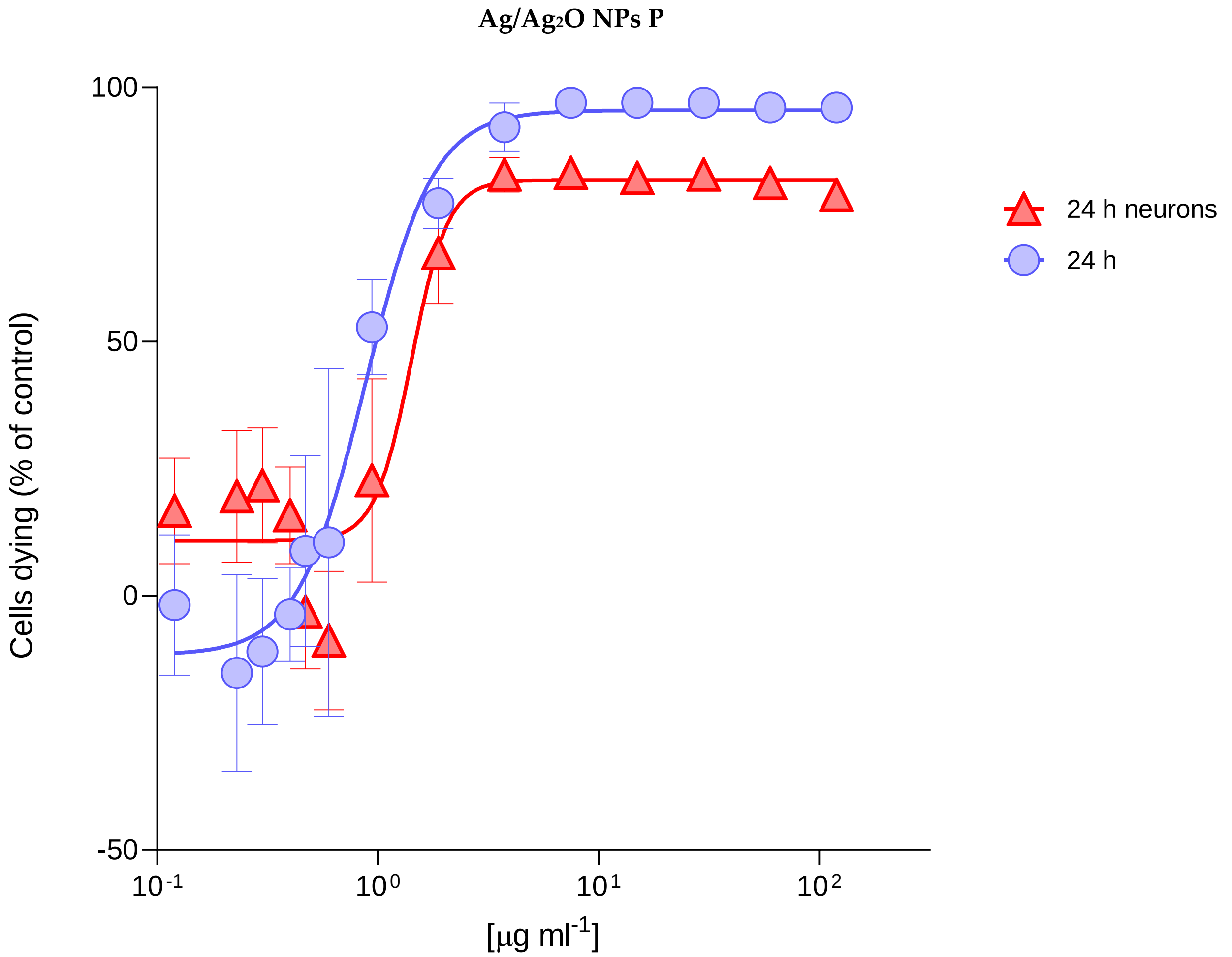

Figure S3. Comparison of Fits analysis reveals significant differences among data sets ($P < 0.0001$), supporting distinct curves for each set ($F(4, 160) = 18.73$).

ZnO NPs A

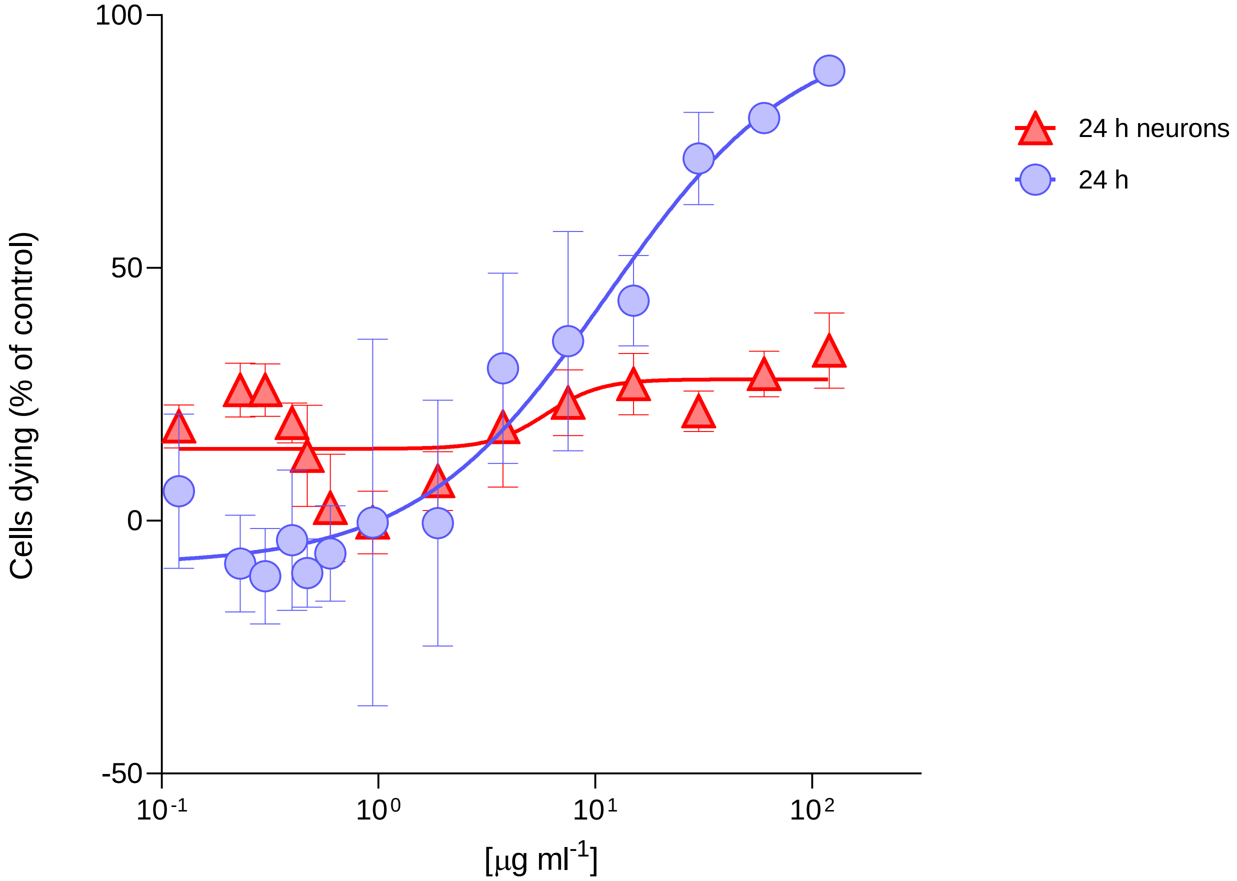

Figure S4. Comparison of Fits analysis shows highly significant differences among data sets ($P < 0.0001$), validating the use of distinct curves for each set ($F(4, 160) = 45.99$).

ZnO NPs P

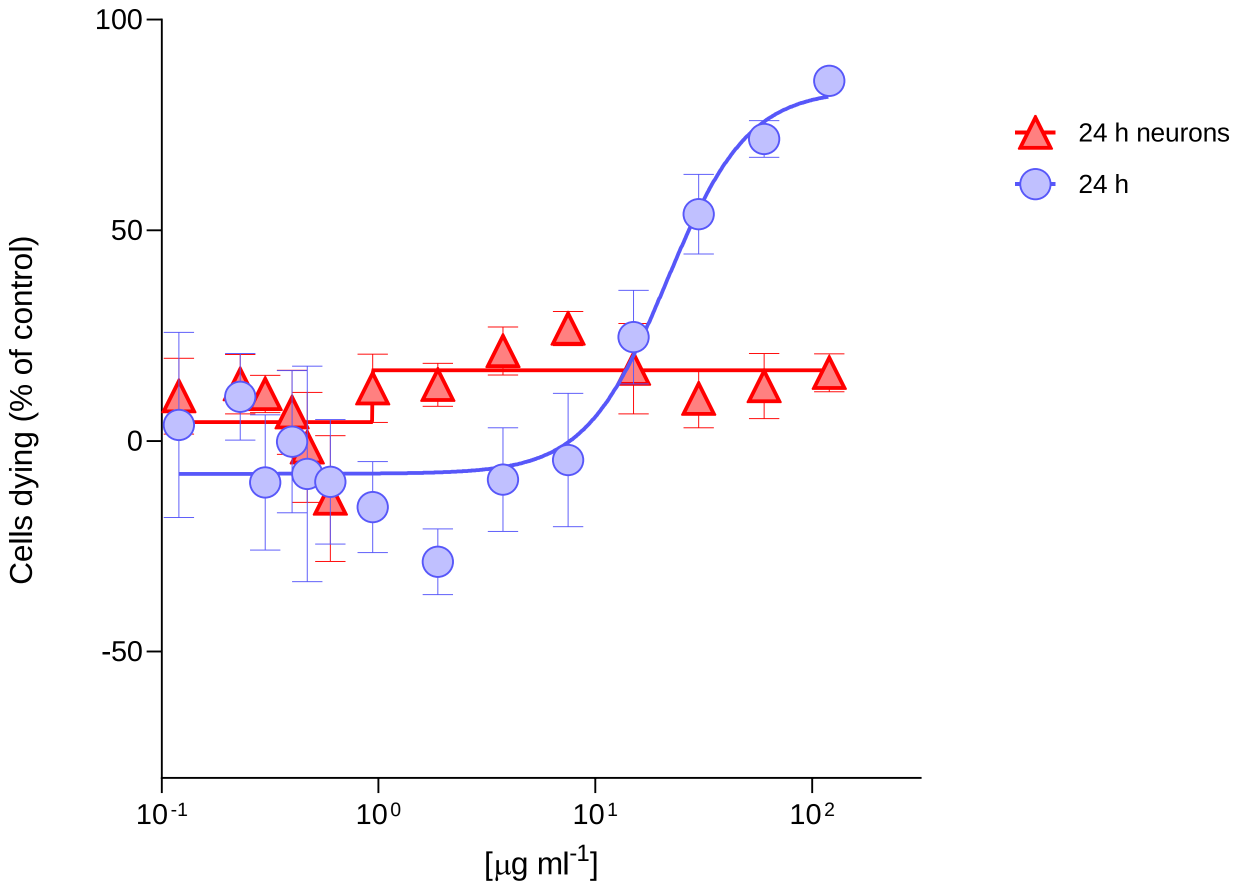

Figure S5. Comparison of Fits analysis confirms significant differences among data sets ($P < 0.0001$), supporting distinct curves for each set ($F(4, 160) = 53.27$).

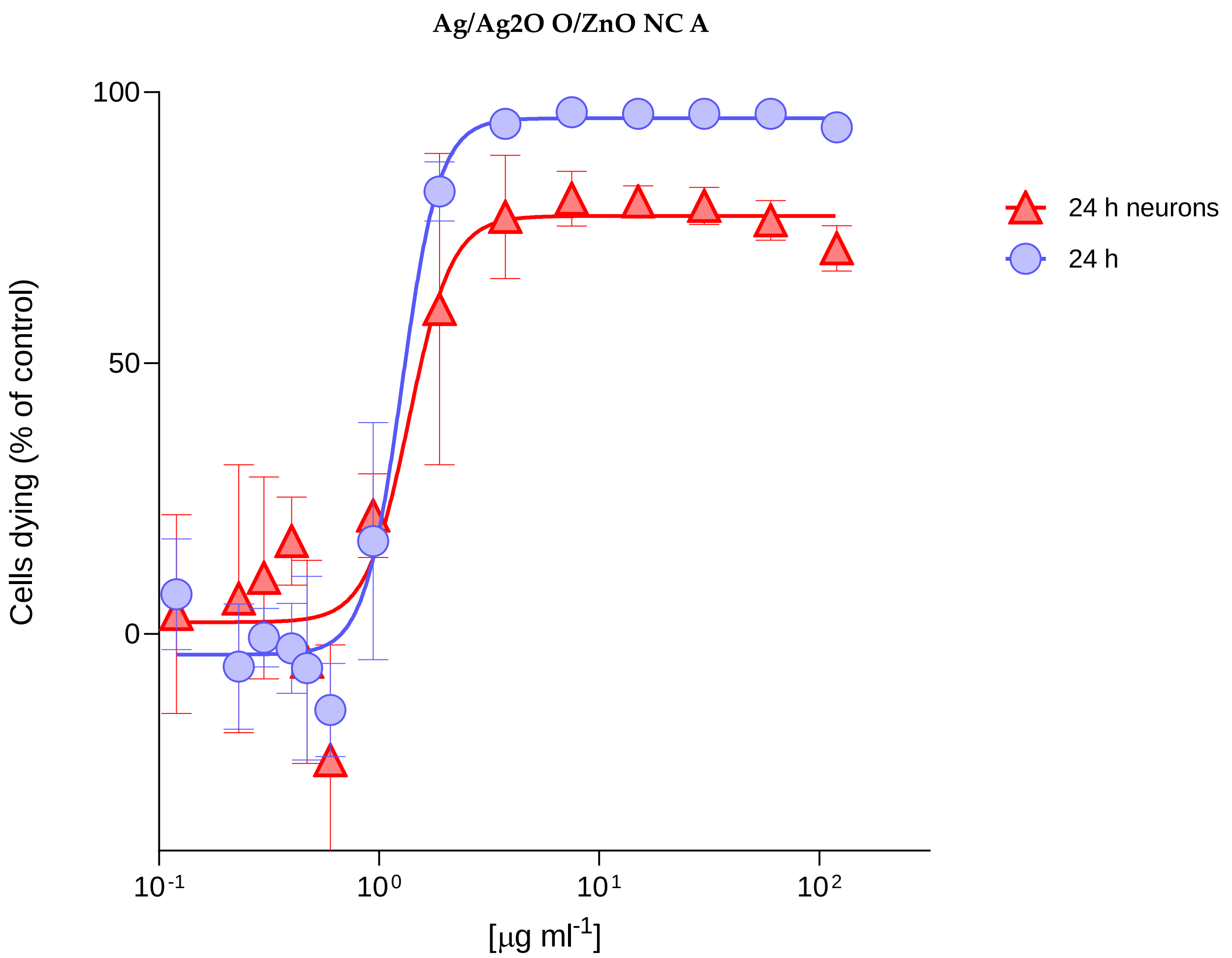

Figure S6. Comparison of Fits analysis demonstrates significant differences among data sets ($P < 0.0001$), favoring distinct curves for each set ($F(4, 160) = 9.831$).

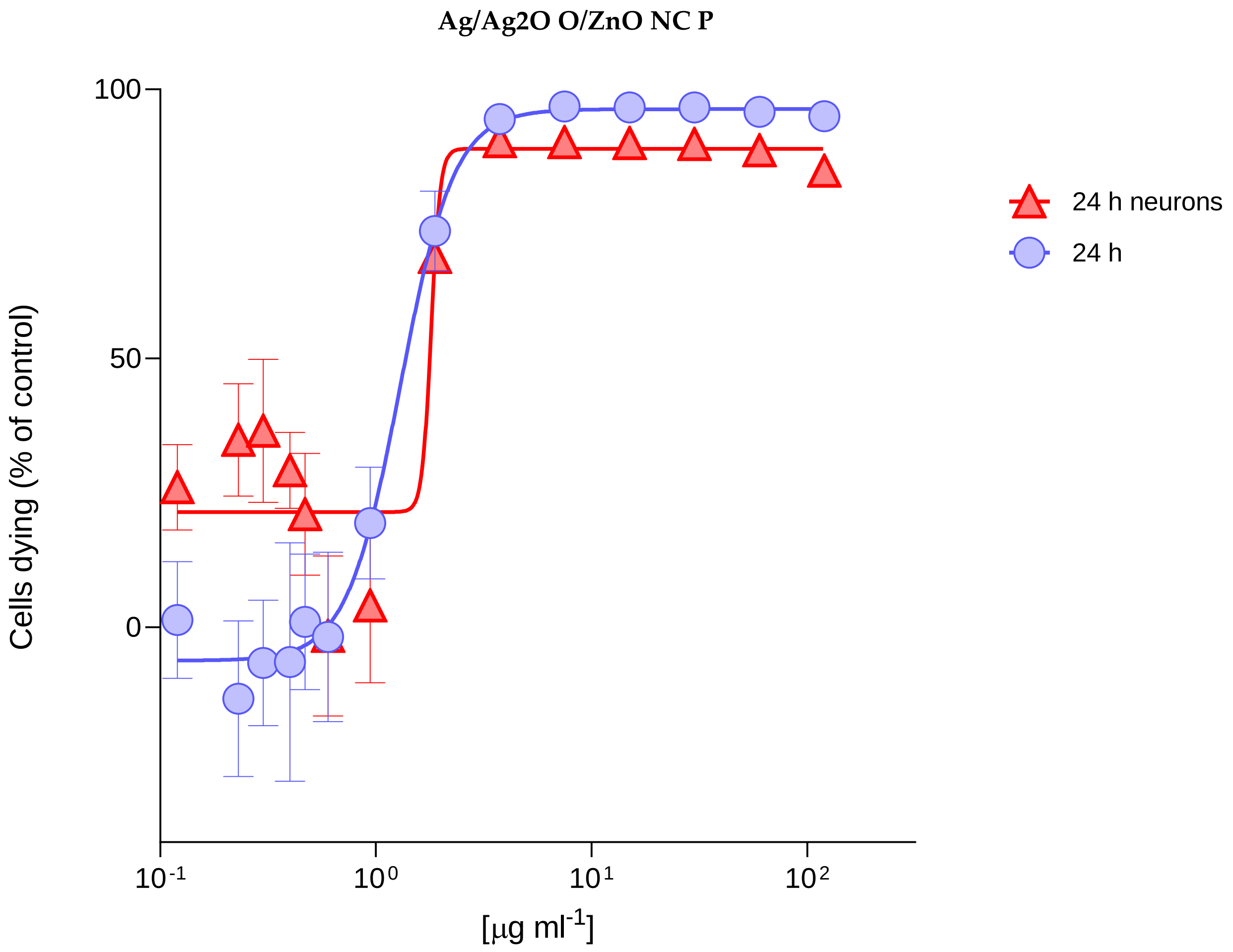

Figure S7. Comparison of Fits analysis reveals significant differences among data sets ($P < 0.0001$), supporting distinct curves for each set ($F(4, 160) = 26.76$).
